# A Comprehensive Review on the Mechanics of Cyclodextrin-Based Slide-Ring Polymers

**DOI:** 10.3390/polym18010037

**Published:** 2025-12-23

**Authors:** D. M. Li, Longyu Wei, Luxi Chen, Bingchang Zhao, Heyang Wei

**Affiliations:** 1 School of Civil Engineering and Architecture, Wuhan University of Technology, Wuhan 430070, China; 2 School of Physics and Mechanics, Wuhan University of Technology, Wuhan 430070, China; 3 Sanya Science and Education Innovation Park, Wuhan University of Technology, Sanya 572000, China; 4 Hubei Key Laboratory of Theory and Application of Advanced Materials Mechanics, Wuhan University of Technology, Wuhan 430070, China; 5 School of Automation, Wuhan University of Technology, Wuhan 430070, China

**Keywords:** slide-ring polymers, topological gels, hyperelasticity, fracture toughness, hysteresis

## Abstract

The widespread application of polymer soft materials in cutting-edge fields such as flexible electronics and biomedicine has placed higher demands on their mechanical properties. Traditional chemically cross-linked or physically cross-linked polymers each have inherent limitations. In contrast, slide-ring polymers (SRPs), also known as sliding cross-linked polymers or topologically cross-linked polymers, effectively distribute chain tension through their slip-cross-link characteristics, thereby exhibiting remarkable toughness, elongation at break, and low hysteresis. Among them, cyclodextrin (CD) has emerged as an ideal building block, such as the CD-based rotaxane/polyrotaxane/pseudortaxane/polypseudortaxane, for constructing SRPs due to its unique cavity structure and ease of modification, enabling diverse regulation of material structure and function through molecular design. Currently, the preparation strategies for cross-linking are relatively well established. However, existing research on the physical and mechanical behavior of SRPs—particularly their responses and damage mechanisms under complex loading conditions—remains unsystematic. Furthermore, establishing a cross-scale correlation mechanism from molecular design to macroscopic performance remains a key challenge. This review systematically summarizes recent advances in the mechanics of cyclodextrin-based sliding cross-linked polymers (CD-based SRPs) focusing on the molecular design and network structures, physical and mechanical behaviors and properties, deformation mechanism and theoretical models, and simulation and prediction, to provide clear guidance for future development of these materials.

## 1. Introduction

Soft materials have emerged as crucial components in cutting-edge fields such as flexible electronics [1,2,3], biomedicine [4,5], and soft robotics [6,7], imposing increasingly demanding requirements on the mechanical properties of polymer materials. Conventional polymers often struggle to meet these demands. Physically cross-linked networks relying on hydrogen bonding or ionic interactions [8,9,10,11,12] typically exhibit insufficient mechanical strength due to weak intermolecular forces, while chemically cross-linked systems [13,14] suffer from random cross-link distribution creating mixed short–long chain networks, where short chains fracture prematurely under stress due to limited deformability [15,16]. In contrast, slide-ring polymers (SRPs) effectively redistribute chain tension through mobile cross-links, achieving exceptional elongation at break (up to 2800%) [17], remarkable toughness (with fracture energy of 3600 J/m^2^) [18], excellent tensile strength (up to 27 MPa), and outstanding low hysteresis (500 cycles without significant hysteresis) [19].

Since de Gennes proposed the “sliding gel” concept in 1999, highlighting its potential for tension equalization [20], subsequent research has sought suitable molecular architectures to realize this theoretical framework. Cyclodextrin (CD) have attracted significant research interest as key building blocks for such systems, owing to their unique amphiphilic cavity structure [21,22,23]. The characteristic “hydrophilic exterior-hydrophobic interior” architecture enables stable pseudorotaxane formation with appropriate polymer chains [24,25]. Furthermore, the abundant surface hydroxyl groups permit diverse chemical modifications, facilitating various sliding cross-link configurations that expand structural diversity and enable multifunctionalization [26,27,28,29]. These attributes establish CDs as ideal constituents for constructing SRPs. Recent advances have demonstrated substantial progress in fabricating CD-based SRPs through precise control of CD functionalization [30,31], polymer backbone selection, and CD variety [32,33], yielding diverse sliding cross-linked networks, also referred to as topological cross-linked networks. This rational molecular design enables precise control over key performance metrics, such as fracture energy, Young’s modulus, and hysteretic dissipation. By establishing a direct bridge from sliding cross-linkers to bulk mechanics, it unlocks the potential for applications that demand exceptional mechanical performance.

Currently, research on the physical and mechanical behavior of SRPs remains insufficiently thorough. Although the large deformation and fracture behavior of classical “figure-of-eight” slide-ring gels have been systematically investigated, SRPs with other cross-linking point configurations have not been fully explored. There is a lack of comprehensive experimental characterization regarding their mechanical responses and damage evolution mechanisms under complex loading conditions. In terms of theoretical modeling, while constitutive models have been developed to a considerable extent, their application in simulation studies has been limited by insufficient experimental validation, resulting in lagging development. Therefore, establishing a cross-scale correlation mechanism—from molecular design to network structure, and further to macroscopic mechanical behavior—has emerged as a crucial research direction for advancing this field.

Although existing reviews have addressed CD-based SRPs, most focus primarily on preparation methods with limited comprehensive analysis [34,35,36,37,38,39,40]. Comprehensive reviews focusing specifically on the mechanics of SRPs remain scarce. To address this gap, this work presents the first systematic review encompassing the cross-linked network structure, physical and mechanical properties, deformation mechanisms, theoretical modeling, and simulation studies of CD-based SRPs. This review systematically examines molecular design strategies, sliding cross-linked network structures, and functionalization of CD-based SRPs (Section 2), comprehensively summarizes their unique mechanical behaviors and properties (Section 3), thoroughly discusses deformation mechanisms and theoretical modeling (Section 4), provides a systematic analysis of simulation studies at different scales (Section 5), and concludes with a summary and perspective (Section 6).

## 2. Cyclodextrin-Based Slide-Ring Polymers

Sliding cross-linking represents a distinctive polymer network strategy wherein the chain segments between cross-links can dynamically adjust their effective length in response to mechanical stress. This capability effectively overcomes the limited deformation capacity of short chains in conventional fixed cross-linked networks, thereby significantly enhancing material toughness, elongation at break, and hysteretic dissipation [41]. Among the various high-strength sliding cross-linked systems developed to date, those based on CD—particularly the “figure-of-eight” cross-linked architecture—constitute one of the earliest successfully demonstrated systems with established reliability [42]. These materials have consequently received considerable research attention and have shown exceptional mechanical properties [43,44,45]. Building upon this foundation, alternative sliding cross-linking configurations have been progressively developed, highlighting the need for a systematic classification of different sliding cross-linking point architectures. The specific geometry of these cross-links and the topological structure of the resulting SRPs networks play a decisive role in governing their deformation mechanisms, ultimately determining their macroscopic physical and mechanical behavior.

### 2.1. Sliding Cross-Linking Point Configurations Based on Cyclodextrin and Their Preparation

CD-based SRPs networks typically employ a pseudorotaxane architecture, wherein linear polymer chains thread through multiple CD molecular cavities. The fundamental challenge in constructing these polymer networks resides in engineering diverse cross-linking configurations through strategic molecular design of CD components. Recent advances have yielded several representative configurations based on distinct cross-linking formation mechanisms. This section will comprehensively examine the structural characteristics, synthetic methodologies, and research progress of these cross-linking configurations, with the objective of establishing fundamental structure–property relationships between microscopic architecture and macroscopic performance.

#### 2.1.1. “Figure-of-Eight” Cross-Linking Point

**Figure 1:**
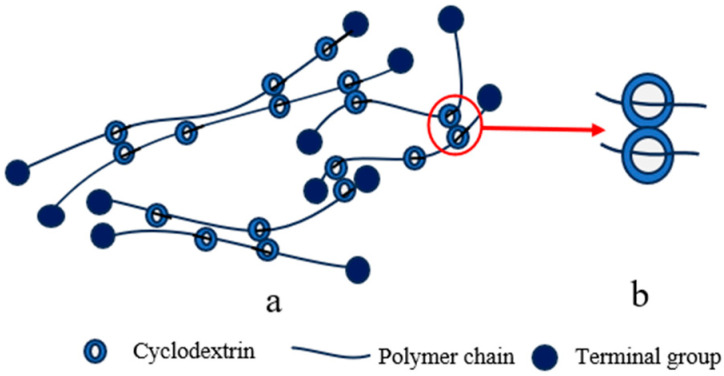
“Figure-of-eight” cross-linking. (**a**) cross-linked network; (**b**) cross-linking point.

In 2001, Ito and colleagues successfully achieved the first synthesis of slide ring (SR) gels, also known as topological gels as a type of SRPs, based on polyrotaxanes (PRs) [42]. Within these systems, the “figure-of-eight” cross-linked structure formed by CD molecules has emerged as one of the most extensively studied and representative core configurations in SRPs. This nomenclature originates from its distinctive cross-linking geometry: bifunctional or multifunctional small molecular cross-linkers serve as bridges, with both ends containing active groups that react with surface functional groups (typically hydroxyl groups) on CDs. This connectivity links CDs on different PR chains into cross-linking points resembling the “figure-of-eight” pattern (Figure 1b), ultimately bridging adjacent PRs into a three-dimensional network (Figure 1a). This unique configuration not only maintains the structural integrity of the polymer network—preventing CD dissociation from the polymer backbone even under substantial deformation—but also permits CD molecules to slide freely along the polymer chains under mechanical stress, effectively distributing localized stress throughout the entire network [42]. These characteristics impart exceptional tensile deformation capability and remarkable toughness to the material [46].

Nevertheless, the synthetic pathway for this cross-linking configuration involves multiple steps and remains cost-intensive. Furthermore, potential dislodgment of the capping groups at PR chain ends [47] may lead to CD slippage from the main chain, ultimately compromising the material’s functional performance.

Despite these limitations, polymers incorporating the “figure-of-eight” cross-linking remain ideal candidates for fabricating high-toughness and high fatigue-resistant elastomers [48]. Subsequently developed representative materials, such as SR gels, have exhibited outstanding performance in experimental characterizations, including high fracture energy (3600 J/m^2^) [18], extreme extensibility (elongation at break up to 2030%) [49], high tensile strength (5.5 MPa) [18], and low hysteretic (showing no significant hysteresis after 500 loading cycles) [19]. These remarkable properties underscore their broad application potential in areas such as flexible devices [50] and biomedical materials [51].

The utilization of small molecule cross-linkers represents the most straightforward approach for constructing such networks. This strategy exploits the multiple hydroxyl groups on CD molecules to react with bifunctional or multifunctional cross-linking agents, thereby forming the characteristic “figure-of-eight” architecture (Table 1). For instance, CDs have been cross-linked through carbonate bonds with *N*,*N′*-carbonyldiimidazole (CDI) [50,51], ether bonds with 1,4-butanediol diglycidyl ether (BDDE) [52] or divinyl sulfone (DVS) [46], and urethane bonds with hexamethylene diisocyanate (HMDI) [53,54,55]. Among these cross-linking chemistries, ether bond formation typically requires alkaline conditions and may involve side reactions, compromising operational simplicity and reproducibility. Urethane bond formation proceeds relatively readily but exhibits sensitivity to moisture, imposing stringent requirements on experimental conditions. Carbonate bonds, while forming under mild conditions with ease, demonstrate comparatively inferior stability.

#### 2.1.2. “Figure-of-Nine” Cross-Linking Point

The “figure-of-nine” cross-linked configuration is characterized by polymer chains threading through CD cavities, while the CD molecules themselves are covalently connected to adjacent polymer chains, thereby establishing a sliding network with distinctive “figure-of-nine” topology. This architecture offers enhanced CD retention and improved material durability. However, its synthesis typically requires meticulous control over CD modification degrees and polymerization conditions, resulting in relatively complex procedures and higher costs.

Despite these challenges, laboratory studies have demonstrated remarkable mechanical properties in such materials. Experimental data reveal exceptional extensibility, with α-Cyclodextrin-Azo hydrogel (αCD-Azo hydrogel) achieving 2800% elongation at break [46] and related elastomers reaching 1632% elongation [56]. These systems simultaneously maintain substantial mechanical strength, exhibiting tensile strength up to 674 kPa [56] and peel strength of 400 N/m (SRPs) [57]. Furthermore, the sliding cross-linked hydrogel demonstrates extraordinary hydration capacity with a swelling ratio of 1800% [37], highlighting its unique network structure and potential for various applications.

**Figure 2:**
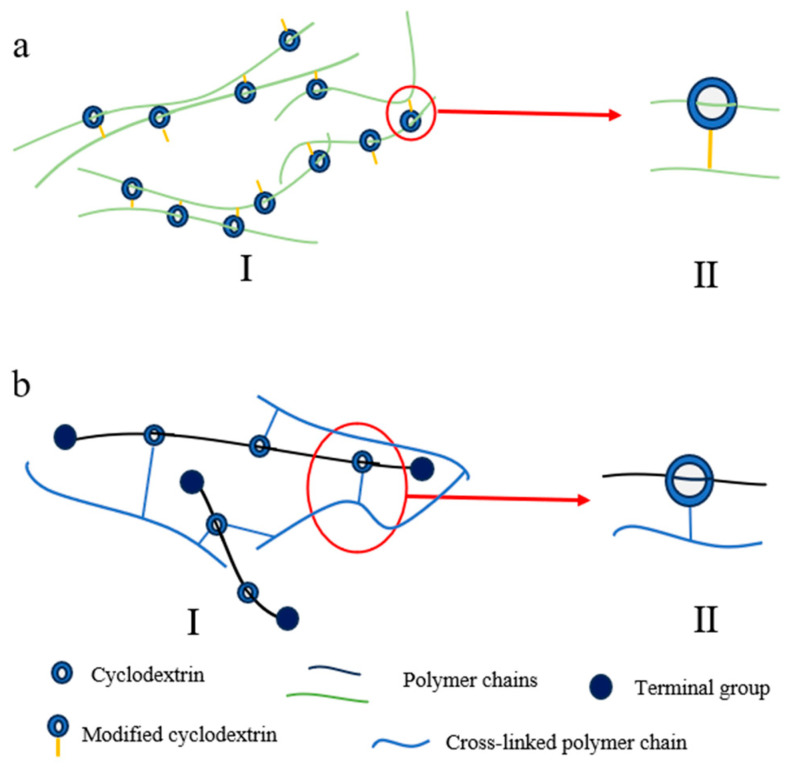
“Figure-of-nine” cross-linked networks and corresponding cross-linking points formed by copolymerization of monomers with (**a**) directly modified CDs; (**b**) modified CDs on PRs.

Two primary synthetic pathways have been developed for constructing these networks. The first approach involves pre-modifying CDs with vinyl or other polymerizable functional groups, followed by direct formation of the sliding cross-linked network through free radical copolymerization with comonomers such as acrylates or isoprene [46,56,57,58] (Figure 2(aII)). The alternative strategy begins with the preparation of PRs, followed by chemical modification of the threaded CDs through hydroxypropylation [59], carboxymethylation [60], or maleic anhydride functionalization [37], ultimately leading to cross-linking reactions with complementary monomers (Figure 2(bII)).

#### 2.1.3. Zipper-Shaped Cross-Linking Points

The zipper-shaped cross-linking configuration features simultaneous inclusion of two polymer chains within gamma-cyclodextrin (γ-CD) cavities, forming a 1:2 host–guest complex that functions as a “molecular zipper” [61,62,63,64]. This architecture offers synthetic simplicity, with certain systems achieving in situ formation of supramolecular cross-links simply through aqueous mixing of components [65,66]. However, this approach presents limitations regarding environmental sensitivity and strong solvent dependence. The swelling behavior of the gel varies with different solvents (such as water, methanol, DMF, and chloroform [66]), which in turn influences the inclusion reaction. Polymerization is conducted under controlled environmental conditions, utilizing either photoinitiated polymerization (under UV irradiation) [66] or redox-initiated polymerization (at low temperatures 0 °C) [66].

Several rotaxane cross-linked polymers (RCPs) based on polytetrahydrofuran (PTHF) [65,66], vinylic supramolecular cross-linkers (VSCs) [67], and polyethylene glycol (PEG) [68] have been successfully developed. Experimental data demonstrate their superior performance compared to conventional CCPs: RCPs exhibit elongation at break up to 1560% (versus 150% for CCPs) and tensile strength of 0.49 MPa (versus 0.10 MPa for CCPs) [65]. Additionally, these RCPs achieve swelling ratios exceeding 2000% in dimethylformamid (DMF) [65,66]. In redox-active applications, SRPs display accelerated deformation rates, with volume change rates threefold higher than conventional hydrogels, alongside 0.92% chemomechanical energy conversion efficiency [67]. Furthermore, other topological network materials have demonstrated a high shape-recovery ratio of 90% under a thermomechanical cycle involving deformation at 120 °C and fixation upon cooling to room temperature [68].

**Figure 3:**
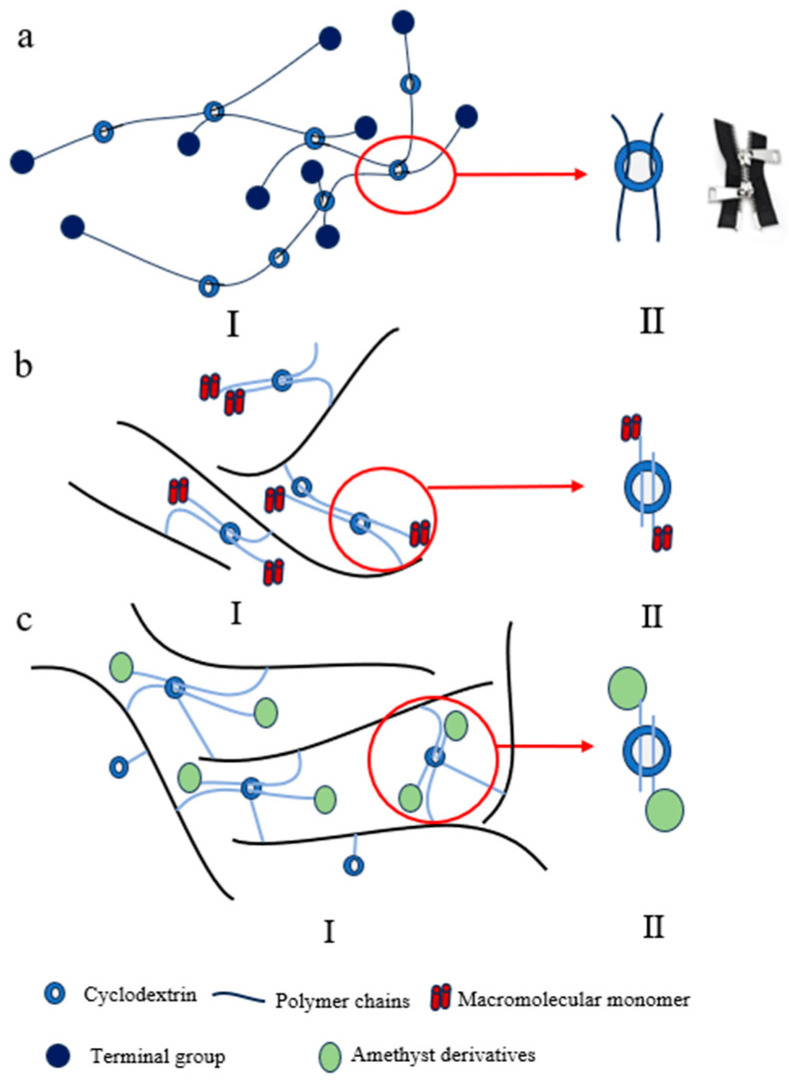
Zipper-shaped cross-linked networks and corresponding cross-linking points prepared by (**a**) physical mixing and self-assembly method; (**b**) in situ formation of supramolecular cross-linkers method; (**c**) direct copolymerization method.

Three primary synthetic pathways have been developed for constructing these networks. The first employs physical mixing and self-assembly [68], where γ-CD and high-molecular-weight PEG are proportionally mixed in aqueous solution. Utilizing the host–guest encapsulation of PEG chains within γ-CD cavities, a zipper-like sliding cross-linked network is formed after end-capping (Figure 3(aII)). The second strategy involves in situ formation of supramolecular cross-linkers [65,66], where γ-CD self-assembles with macromolecular monomers bearing bulky end-groups in aqueous media to create VSCs. These are subsequently incorporated into polymer networks via free radical polymerization with vinyl monomers (Figure 3(bII)). The third approach utilizes direct copolymerization [67], wherein γ-CD and guest molecules (such as viologen with C11 alkyl chain, VC11) are grafted onto polymer backbones separately, enabling direct construction of polymer networks through host–guest inclusion interactions (Figure 3(cII)).

#### 2.1.4. Fidget Spinner-Shaped Cross-Linking Points

The fidget spinner-shaped cross-linking configuration is characterized by multiple covalently linked CD molecules threading onto polymer main chains to form sliding cross-links. This architecture enables simultaneous cross-linking of more than two polymer chains, though it presents challenges including complex synthesis and pronounced sensitivity to component concentrations [69]. Corresponding SR gels exhibiting high extensibility (up to 1200% elongation at break) [70]. Furthermore, when SR hydrogels were incubated with cell culture medium, cell viability remained nearly 100%, confirming excellent biocompatibility [71].

**Figure 4:**
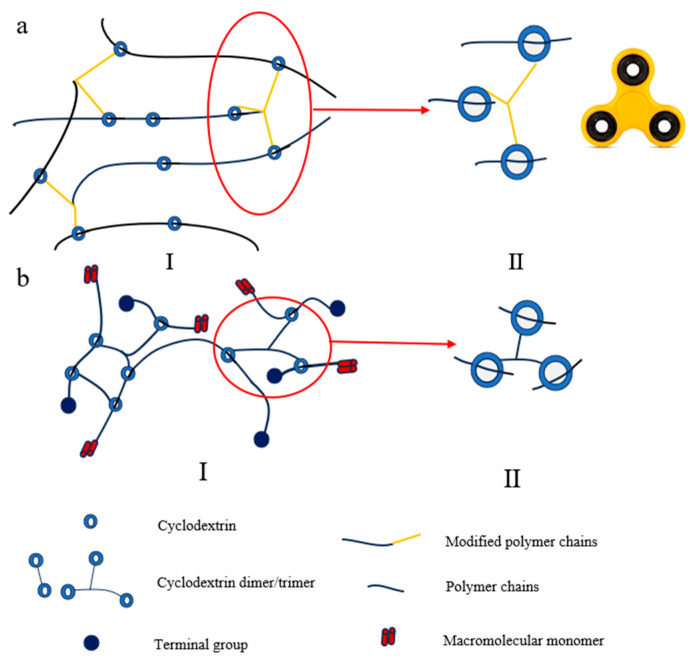
Fidget spinner-shaped cross-linked networks and corresponding cross-linking points prepared by (**a**) azidated CD mixed with PEG forms a cross-linked network; (**b**) CD dimers or trimers mixed with PEG form a cross-linked network.

Two synthetic routes have been developed for constructing fidget spinner-type cross-linked networks. The first approach involves introducing azide groups onto CD molecules, which are subsequently mixed with dialkynyl-functionalized PEG. Through click chemistry, this system simultaneously constructs the rotaxane architecture and establishes sliding cross-links, with CD serving dual roles as both capping agent and cross-linking moiety (Figure 4(aII)). The alternative method employs pre-synthesized CD dimers or trimers prepared via click chemistry, which then self-assemble in aqueous solution with PEG macromers containing bulky end-groups through host–guest interactions. This process forms fidget spinner-like cross-linking points that ultimately generate the sliding cross-linked polymer network (Figure 4(bII)).

#### 2.1.5. Crystal Domain-Type Cross-Linking Points

**Figure 5:**
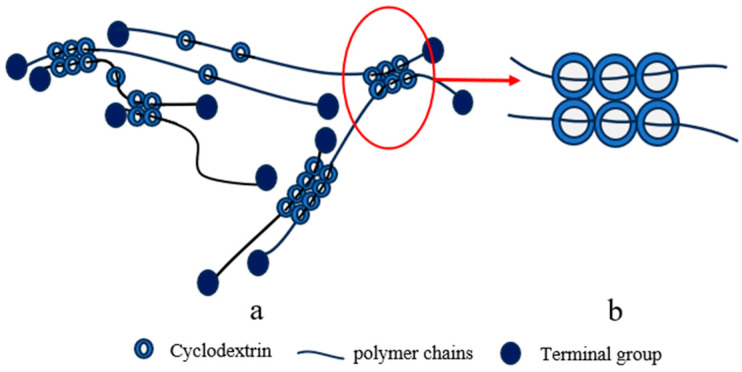
Crystal domain-type cross-linking. (**a**) cross-linked network; (**b**) cross-linking point.

The crystal domain-type cross-linking configuration is established when multiple PEG chains threaded by numerous CD molecules form PRs, where the CD-incorporated segments subsequently self-assemble into ordered columnar nanocrystals through hydrophobic interactions. These microcrystalline domains function as physical cross-links that stabilize the flexible PEG network (Figure 5b), thereby creating a crystal domain-cross-linked architecture [72]. This configuration offers straightforward material preparation as its primary advantage, though it suffers from significant pH sensitivity during cross-linking and limited cross-linking efficiency. Structural characterization of hydrogels prepared with this configuration has been conducted through helium adsorption and X-ray diffraction analyses, while comprehensive mechanical property evaluation remains to be systematically investigated.

#### 2.1.6. Dumbbell-Shaped Cross-Linking Points

**Figure 6:**
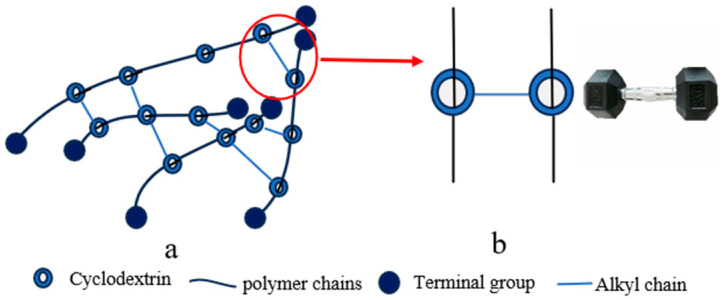
Dumbbell-shaped cross-linking. (**a**) cross-linked network; (**b**) cross-linking point.

The dumbbell-shaped cross-linking configuration is characterized by the covalent linkage between CDs previously threaded onto a polymer backbone, typically achieved through reaction with alkyl-based cross-linkers to form a sliding network (Figure 6b). This architecture provides an enhanced sliding range for the cross-links, though it requires multi-step synthesis with precisely controlled reaction conditions and suffers from relatively low yields [73]. Current research on such materials remains primarily proof-of-concept, with mechanical properties yet to be fully explored. A representative synthetic strategy involves forming an inclusion complex between polypropylene oxide (PPO) and CD, followed by cross-linking with alkyl agents under alkaline conditions [73]. Experimental characterization demonstrates favorable thermal stability with network integrity maintained below 200 °C, alongside a swelling ratio by volume of 4 [73].

#### 2.1.7. Tanghulu-Shaped Cross-Linking Points

**Figure 7:**
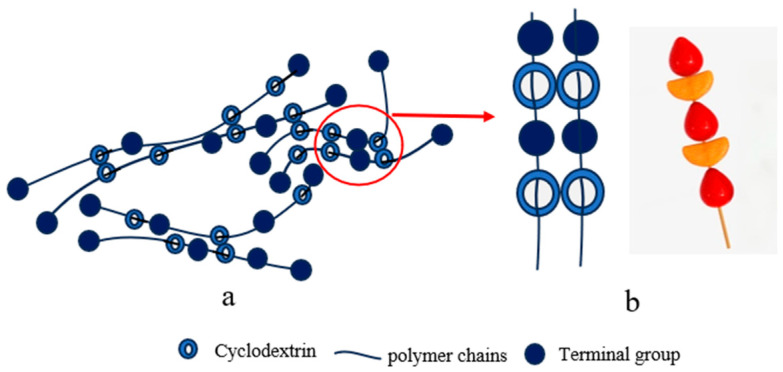
Tanghulu-shaped cross-linking. (**a**) cross-linked network; (**b**) cross-linking point.

The tanghulu-shaped configuration features multiple stopper sites introduced at regular intervals along the PR backbone (Figure 7b), effectively dividing the long chain into independently sliding segments. This design alters the material’s failure mechanism from conventional “whole-chain fracture” to “localized segment failure”, significantly improving gel durability. However, excessive stopper incorporation restricts CD mobility, reducing ultimate strength and potentially introducing additional fracture points. Currently, this strategy remains primarily in the simulation stage. Through coarse-grained molecular dynamics simulations, Li and Liu [47] demonstrated that while maintaining high ductility (elongation at break of 53%), this configuration significantly delays stress collapse through controlled strength sacrifice, thereby enhancing fracture toughness and service life.

### 2.2. Hybrid Cross-Linking

To address the limitations of single-network systems—such as functional constraints and competing property trade-offs—researchers have developed hybrid cross-linking strategies. A good example for hybrid cross-linking strategy is the work by Suo et al. [74], who used a small amount of chemical cross-linking combined with a large amount of physical entanglement to form hybrid cross-linked polymers, achieving both high strength and high fracture toughness. In the hybrid cross-linked network, the sparse chemical cross-linking maintains the network integrity, while the abundant physical entanglements achieve an effect of balancing chain lengths as the slide-ring cross-linking, thereby improving the mechanical properties.

A slide-ring polymer network incorporating multiple types of slide-ring cross-links essentially constitutes a hybrid slide-ring/slide-ring system. Hybrid slide-ring/chemical polymers incorporate both sliding cross-links and covalent cross-links, typically achieved by covalently fixing CDs or polymer chains of PRs within the polymer matrix. In such systems, PRs participate in copolymerization as cross-linking agents.

**Figure 8:**
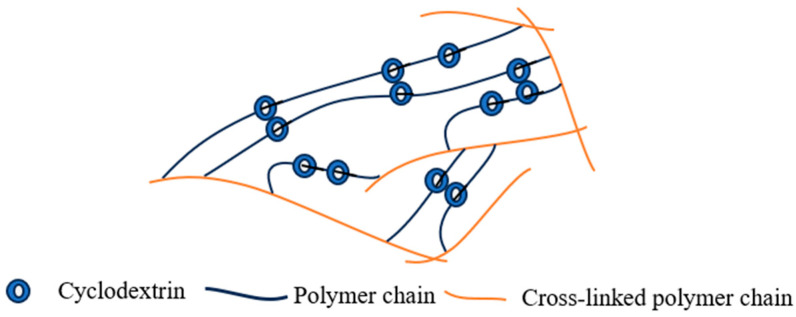
Hybrid network of topological cross-linking and chemical cross-linking.

Wang et al. [75] constructed a covalent network via photoinitiated radical polymerization of acrylamide (AM), during which PRs were incorporated, resulting in a hybrid cross-linked hydrogel (Figure 8). This SR supramolecular hydrogel exhibited high extensibility (elongation at break of 2540%), high toughness (fracture work of 17.4 MJ/m^3^), and low hysteretic (plastic strain stabilized below 10% after 10 cycles at 1100% strain). Zhang et al. [76] copolymerized PRs with AM and methacrylate (MA) monomers, embedding the PRs within the covalent network. The resulting hydrogel demonstrated excellent self-recovery, with storage (G′) and loss (G″) moduli returning to their initial states after repeated large-strain failure and small-strain recovery cycles, though mechanical property data remain unreported. Dikshit et al. [77] combined PRs with lipoic acid monomers, forming sliding cross-links via CD–monomer reactions and covalent cross-links through disulfide bonds between lipoic acid units. The resulting material showed remarkable load-bearing capacity (100 mg of adhesive lifting 4 kg weight) and aging resistance (no significant bond strength loss after four weeks).

Hybrid slide-ring and physical cross-linked polymers integrate CD-based sliding cross-links with supplementary physical interactions within a unified system. In such networks, polymer main chains thread through multiple CD cavities to form PRs, simultaneously, through non-covalent interactions; additional physical cross-links are formed within the slide-ring network, thereby constructing a hybrid network architecture that incorporates both sliding cross-links and physical cross-links.

**Figure 9:**
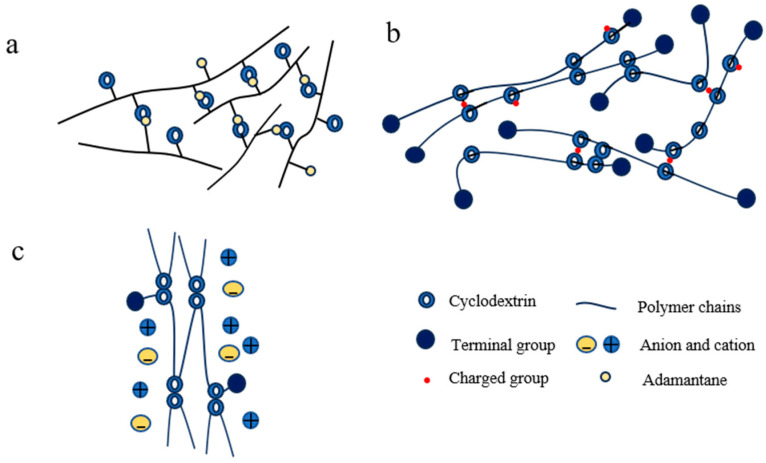
Hybrid network of topological cross-linking and physical cross-linking prepared with (**a**) the inclusion complexation between CDs attached to side chains forms cross-linked network; (**b**) SR-PC network; (**c**) sliding cross-linked network using ionic liquids.

Kimura et al. [78] developed hybrid network by threading polymer chains through β-Cyclodextrin (β-CD) to form rotaxane structures while grafting additional β-CD units to side chains for host–guest complexation with adamantane (Figure 9a). The resulting gel exhibited notable stress relaxation behavior. Tang et al. [79,80] employed molecular dynamics simulations to design SR-PC hybrid networks, where charged CDs in PRs formed electrostatic physical cross-links (Figure 9b). The simulated SR-PC gels showed enhanced strength (approximately 10% higher than conventional covalently cross-linked gels) and superior fatigue toughness (half the cyclic fatigue damage of FC gels). Enoki et al. [81] modulated a sliding cross-linked network using ionic liquids, where strain-induced crystallization created reversible physical cross-links during deformation (Figure 9c). The resulting SR ionic gel demonstrated high toughness (fracture work of 48 MJ/m^3^) and substantial ionic conductivity (approximately 2 mS/cm at 313 K).

### 2.3. Functionalization of CD-Based SRPs

CD-based SRPs demonstrate distinctive advantages that extend beyond their exceptional mechanical properties to encompass enhanced multifunctionality. This versatility primarily stems from two structural characteristics of CDs: their readily modifiable molecular architecture and the adaptable nature of the polymer backbone threading through their cavities. Table 2 summarizes the implementation methods and recent progress in functionalization of CD-based SRPs.

**Table 2:** Functional CD-based SRPs.

Function	Implementation Method	Research Progress
Thermal function	polymer main chain poly(*N*-isopropylacrylamide) PNIPA [59], methylated CD [48]	reversible swelling and contraction [59], thermoresponsive behavior regulated by Ph [59] (Figure 10a), thermal stability [82], thermoresponsive characteristics [48].
Electrical function	choline chloride (ChCl) [19], functionalized CD [83]	PR content affects conductivity [83] (Figure 10b), excellent ionic conductivity (0.93 S/m) [19], sensitive resistance response to strain (Figure 10c), stable signal output [19] (Figure 10d).
Optical function	azobenzene groups into β-CD	rapid photoresponsive behavior [17] (Figure 10e), sensitive optical signal response [58] (Figure 10f),
Acoustic function	internal friction effect, internal porous structure.	excellent damping performance [84] (Figure 10g).

**Figure 10:**
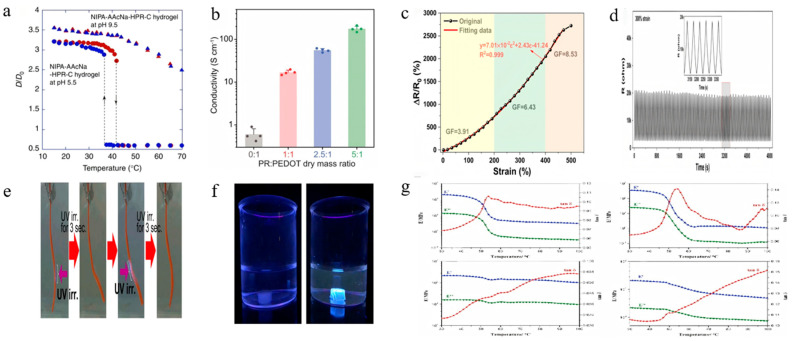
Functionalization of CD-based SRPs. (**a**) Variation in equilibrium swelling degree of functionalization with SRPs with temperature (Reproduced from Ref. [59] with permission from Springer Nature). (**b**) Effect of PR content on the film conductivity (Reproduced from Ref. [83] with permission from The American Association for the Advancement of Science). (**c**) Relative resistance changes in the PR-Gel as a function of tensile strain (Reproduced from Ref. [19] with permission from Springer Nature). (**d**) Relative resistance response of PR-Gel over 100 cycles at 300% strain (Reproduced from Ref. [19] with permission from Springer Nature). (**e**) Rapid and reversible bending of the αCD-Azo xerogel in response to UV light (Reproduced from Ref. [17] with permission from American Chemical Society). (**f**) Photographs of gels before and after ultraviolet irradiation (Reproduced from Ref. [58] with permission from Springer Nature). (**g**) Enhanced damping performance of polyurethanes cross-linked by the slide-ring with β-CD [3] PR (Reproduced from Ref. [84] with permission from Elsevier).

## 3. Physical and Mechanical Behaviors and Properties of CD-Based SRPs

CD-based SRPs demonstrate distinct mechanical behavior compared to CCPs at both the macro and micro levels, attributable to their unique sliding cross-linked architecture [85,86,87,88,89,90,91]. The mobility of sliding cross-links under external load enables redistribution of chain tension, effectively delaying microscopic damage accumulation and profoundly influencing material deformation response, fracture progression, and fatigue lifetime. This chapter systematically reviewed the state-of-art literatures on the investigation of the mechanical properties of SRPs under various loading conditions, with particular focus on their mechanical response during tensile, viscoelastic, and multiaxial deformation, aiming to elucidate the regulatory role of the sliding cross-linked structures and their underlying mechanisms in determining the macroscopic material properties.

### 3.1. Deformation

The fundamental mechanism of SRPs originates from the sliding capability of their cross-links. Under tensile, viscoelastic, and multiaxial deformation, this unique architecture enables adaptive reorganization in response to varied stress fields, achieving exceptional elongation at break, remarkable toughness, and outstanding low hysteresis. These observations establish a clear structure–property relationship between microscopic sliding dynamics and macroscopic mechanical behavior.

#### 3.1.1. Stretching Deformation

Experimental investigation of the tensile properties of CD-based SRPs has revealed distinctive mechanical behaviors that fundamentally differ from those of fixed cross-linked polymers. Uniaxial tensile testing, as the primary characterization method, typically employs specimens prepared through capillary injection, mold casting, or film formation [51,66], with common geometries including rectangular strips and dumbbell shapes [46,65]. Mechanical testing is predominantly conducted in ambient air at room temperature, though some experiments are performed in solution environments [48,52,90,92]. Specimen thickness generally ranges from 0.5 mm to 5 mm, with tests performed on tensile testing machines at crosshead speeds between 0.0083 mm/s and 1.875 mm/s. Table 3 summarizes the size, type, and tensile rate of the experimental investigations.

**Figure 11:**
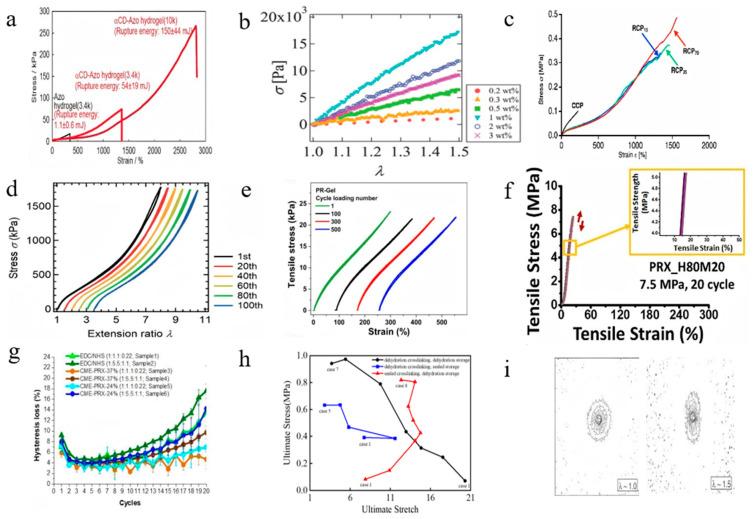
Tensile deformation. (**a**) Stress–strain curves of αCD-Azo hydrogels prepared with different PEG molecular weights (Reproduced from Ref. [17] with permission from American Chemical Society). (**b**) Stress–strain curves at different concentrations of cross-linking agents (Reproduced from Ref. [51] with permission from Royal Society of Chemistry). (**c**) Stress–strain curves of RCPs prepared with different main chain lengths (Reproduced from Ref. [65] with permission from Elsevier). (**d**) Stress–strain curves under 100 loading cycles of SR gel, with a PEG volume fraction of 38% (Reproduced from Ref. [18] with permission from The American Association for the Advancement of Science). (**e**) Stress–strain curves under 500 loading cycles of SR gel, with AM concentration of 40 *w*/*v*% (Reproduced from Ref. [19] with permission from Springer Nature). (**f**) Stress–strain curves under 20 loading cycles of PR elastomer (Reproduced from Ref. [94] with permission from Elsevier). (**g**) Hysteresis loss during 20 loading cycles of SRPs with different coverage rate (Reproduced from Ref. [96] with permission from MDPI). (**h**) Ultimate stress vs. ultimate stretch of the SR gels with 3 kinds of dehydration processes (Reproduced from Ref. [49] with permission from Wuhan University of Technology). (**i**) The normal butterfly pattern of the sliding gel in small-angle X-ray scattering (Reproduced from Ref. [50] with permission from Elsevier).

Extensive experimental results demonstrate that exceptional tensile performance represents one of the most notable characteristics of SRPs, and this performance can be effectively modulated through molecular structure parameters. In studies utilizing rectangular strip specimens, SRPs have achieved progressive improvements in tensile strength of 5.5 MPa (SR gel with PEG volume fraction of 38%) [18] and elongation at break up to 2030% (SR gel with a water content of 40.63%) [49]. The specific experimental data from different studies are shown in Table 4. Dumbbell-shaped specimens similarly confirm extraordinary ductility, with tensile strength of 0.49 MPa [65] and elongation at break up to 2800% (αCD-Azo hydrogel) [17] (Figure 11a).

Further studies have indicated that the characteristics and concentration of the cross-linker are key parameters for enhancing the mechanical properties of SRPs. Reducing cross-linker concentration (e.g., from 3 wt% to 0.2 wt%) induces an unusual initial increase followed by decrease in the elastic modulus of SR gels, effectively enhancing elastic modulus to 40 kPa [51] (Figure 11b). According to Mayumi et al. [51], the anomalous concentration dependence of the elastic modulus in SR gels originates from two distinct regimes governed by cross-linking density. At low cross-linking density, high fraction of uncross-linked CDs restricts the sliding motion of cross-linking points. In this regime, elasticity is primarily governed by the conformational entropy of the polymer chains, leading to an increase in the modulus with increasing cross-linking density. At high cross-linking density, however, CDs are extensively consumed, diminishing their topological constraint effect. This results in weakening of the entropic contribution and an enhanced freedom for cross-linking points to slide, ultimately causing a decrease in the modulus. Increasing cross-linker molecular weight (PEG from 20 kDa to 100 kDa) elevates both elongation at break (from 500% to 800%) and tensile strength (from 48 kPa to 120 kPa) in PNIPA gels at equivalent cross-linking density [93]. Similarly, increasing PEG molecular weight from 3.4 k to 10 k significantly enhances the elongation at break of αCD-Azo hydrogel from 1350% to 2800% [17] (Figure 11a). Studies also indicate that longer polymer backbones improve mechanical performance: in RCPs, increasing PTHF chain length from 15 to 70 units raises elongation at break from 1340% to 1560% and tensile strength from 0.334 MPa to 0.49 MPa, respectively [65] (Figure 11c).

In cyclic loading-unloading tests, SRPs exhibit minimal low hysteresis and nearly complete elastic recovery. SR gels demonstrate approximately 100% rapid reversible deformation over 100 cycles, indicating exceptional low hysteresis [18] (Figure 11d). After five loading cycles to 100% strain, permanent deformation remains below 3%, while SR gel with AM concentration of 40 *w*/*v*% shows no significant hysteresis after 500 cycles at 300% strain [19] (Figure 11e). Elastomeric materials similarly perform well in cyclic mechanical tests. Jang et al. [66] noted that RCPs maintain high elongation at break alongside exceptional low hysteresis, while Seo et al. [94] conducted 20-cycle tensile tests on PR elastomers, with low hysteretic of 1.5% (Figure 11f). Ikura et al. [95] observed maintained response stability in SRPs with KB mass fraction of 10% through 100 cycles at 100% strain. Lee et al. [96] demonstrated that cross-linking gelatin hydrogels with threading α-CDs exhibit relatively low hysteresis (at the level of 10–15%) over 20 loading cycles, significantly outperforming conventional chemically cross-linked hydrogels (Figure 11g). Table 4 summarizes the data of mechanical properties of CD-based SRPs under uniaxial tension.

**Table 3:** Uniaxial tensile test.

Experimental Conditions	
Rectangular strip	15 × 2 × 1 mm^3^ [18], 10 × 2 × 0.5 mm^3^ [51], 60 × (6–8) × (1.2–5) mm^3^ [52], 16 × 4 × 0.5 mm^3^ [70], 15 × 10 × 1 mm^3^ [56], 30 × 5 × 0.5 mm^3^ [59], 10 × 5 × 1 mm^3^ [66], 10 × 4 × 1 mm^3^ [93], 30 × 3 ×1 mm^3^ [49,97].
Dumbbell shape	12 × 2 × (0.68–0.94) mm^3^ [65], 10 × 2 × 1 mm^3^ [96], 20 × 5 × 1 mm^3^ [95].
Rate	0.0083 mm/s [93,94], 0.1 mm/s [51,56,59,96], 0.16 mm/s [65], 0.53 mm/s [70], 1 mm/s [10,17,95], 1.67 mm/s [19,49,52,66,97], 1.875 mm/s [18].

**Table 4:** Mechanical property of CD-based SRPs under uniaxial tension.

Uniaxial Tension	
Elongation at break	200% (SRPs mixed with HDI and MDI in an 80:20 ratio) [94], 450% (RCPs with TBM-2 cross-linking agent) [66], 800% (PNIPA gel) [93], 830% (SR gel with AM concentration of 40 *w*/*v*%) [19], 991% (SR gel with coverage rate of 4.4%) [97], 1200% (SR gel with molecular weight of 35 kDa of PEG) [70], 1480% (SRPs with ion cross-linking agent concentration of 0.8 wt%) [59], 1530% (SR gels with PEG volume fraction of 18%) [18], 1560% (RCPs with degree of polymerization (DP*n*) value of 70 for PTHF parts) [65], 1650% (SRPs with cross-linking agent concentration of 5 w%) [56], 2030% (SR gel with water content of 40.63%) [49], 2800% (αCD-Azo hydrogel) [17].
Tensile strength	40.9 kPa (SRPs with cross-linking agent concentration of 2 wt%) [59], 78.1 kPa (SR gel with AM concentration of 40 *w*/*v*%) [19], 130 kPa (SR gel with molecular weight of 100 kDa of PEG) [70], 270 kPa (αCD-Azo hydrogel) [17], 444 kPa (SR gel with coverage rate of 4.4%) [97], 490 kPa (RCPs with DP*n* value of 70 for PTHF parts) [65], 674 kPa (SRPs with cross-linking agent of 1 w%) [56], 974 kPa (SR gel with water content of 18.36%) [49], 5.5 MPa (SR gel with PEG volume fraction of 38%) [18], 8.7 MPa (SRPs mixed with HDI and MDI in an 80:20 ratio) [94], 27 MPa (RCPs with TBM-2 cross-linking agent) [66].
Elastic modulus	44.4 Pa (RCPs with DP*n* value of 70 for PTHF parts) [65], 0.32 kPa (SRPs with coverage rate of 24%) [96], 8.2 kPa (SR gel with volume swelling rate of 12.1) [52], 17 kPa (SR gel with cross-linking concentration of 1%) [51], 26.7 kPa (SR gel with molecular weight of 35 kDa of PEG) [70], 30 kPa (PNIPA gel) [93], 109 kPa (SR gel with coverage rate of 4.4%) [97], 130 kPa (SR gels with PEG volume fraction of 18%) [18], 668 kPa (SR gel with water content of 17.5%) [49].
Low hysteresis	After 20 cycles of tensile testing, PR elastomer demonstrated excellent elastic recovery ability with low hysteresis [94]. After 20 loading cycles, gelatin hydrogel exhibited relatively low hysteresis, ranging from 10% to 15% [96]. After 100 cycles at 100% strain, SRPs with KB mass fraction of 10% still maintained good response stability [95]. After 100 cycles at 800% strain, SR gels with PEG volume fraction of 38% demonstrated nearly complete rapid reversible deformation [18]. After 500 cycles at 300% strain, SR gel with AM concentration of 40 *w*/*v*% almost no significant hysteresis [19].

Li and Fu [49] prepared SR gel with different water content through three distinct dehydration strategies and conducted uniaxial tensile tests, revealing that dehydrated SR gels exhibit higher fracture resistance with elongation at break of 2030% (SR gel with water content of 40.63%), elastic modulus of 668 kPa (SR gel with water content of 17.5%), and tensile strength of 0.974 MPa (SR gel with water content of 18.36%) (Figure 11h) than their hydrated counterparts. More importantly, the hydration–dehydration cycles achieve a completely reversible mechanism of deformation, that is, the transition between I_2_-related and I_2_-independent hyperelasticity [49]. Through the fabrication of SR gel with varying coverage and cross-linking densities followed by uniaxial tensile testing, Li and Zhang [97] demonstrated that the SR gel sample with a coverage rate of 4.4% exhibits superior mechanical properties with elongation at break of 991%, tensile strength of 0.444 MPa, and elastic modulus of 109 kPa.

The influence of the sliding cross-linked structure of SRPs on their macroscopic mechanical behavior can be directly observed through in situ structural characterization techniques such as small-angle X-ray/neutron scattering. During stretching, SRPs display a unique “normal butterfly pattern”, contrasting sharply with the “abnormal butterfly pattern” observed in conventional gels (Figure 11i), indicating that sliding cross-links uniformly distribute stress and effectively suppress microstructural heterogeneity [50,59,98]. Furthermore, mechanical behavior changes induced by solvent environment variation (good solvent NaOH versus poor solvent water) [90] or cross-linking density adjustment (0.2 wt–3 wt%) [51] provide additional confirmation of the sliding mechanism and its dominant role.

In summary, tensile testing and structural characterization collectively demonstrate that the sliding cross-linking mechanism fundamentally enables SRPs to achieve ultrahigh extensibility, low hysteresis, and exceptional low hysteresis.

#### 3.1.2. Viscoelasticity

Research on the viscoelastic characteristics of CD-based SRPs has demonstrated that their sliding cross-linking architecture imparts unique dynamic mechanical behavior and energy dissipation mechanisms. Dynamic mechanical analysis (DMA) serves as the primary experimental technique for investigating SRPs viscoelasticity, typically employed to examine their viscoelastic evolution under small-amplitude oscillatory shear (SAOS) conditions by applying a sinusoidal shear strain and performing frequency sweeps [53]. According to Kato and Ito [53], the viscoelastic characteristics of SR gels arise from their distinctive slide cross-linked network architecture. On short time scales, the cross-links act as fixed points, and the material displays conventional rubber-like elasticity. Over longer time periods, the sliding of cross-links permits the relaxation of partial chain segments, thereby relieving segmental orientational anisotropy. This leads to a dynamic transition from a rubbery state to a sliding state.

**Figure 12:**
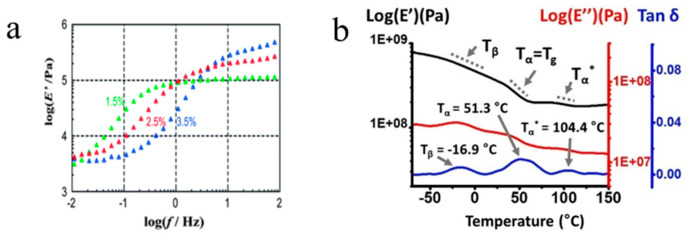
Viscoelastic deformation. (**a**) Viscoelastic relaxation of SR gels with different concentrations of cross-linking agents (Reproduced from Ref. [53] with permission from Royal Society of Chemistry). (**b**) Behavior of SRPs at different temperatures (Reproduced from Ref. [94] with permission from Elsevier).

The viscoelastic behavior of these materials originates fundamentally from the mobility of their sliding cross-links. Kato and Ito [53] provided the first direct experimental evidence through DMA of a unique dynamic transition in SR gels, identifying two distinct plateau regions in the frequency spectrum: a rubbery plateau at high frequencies and a sliding elasticity plateau at low frequencies (Figure 12a). The pronounced relaxation between these plateaus—characterized by a modulus decrease of nearly two orders of magnitude—was attributed to the diffusive sliding of polymer chains through cross-linking points. Seo et al. [94] observed through DMA that PR elastomers display typical semi-crystalline elastomer behavior, including a crystal–crystal slip transition temperature associated with non-cross-linked crystalline domains (Figure 12b). This rate-dependent behavior reflects the distinctive viscoelastic and energy dissipation mechanisms enabled by sliding cross-linking.

This analysis elucidates the fundamental mechanism underlying the viscoelastic behavior of SRPs: the observed dual-plateau response originates from the sliding capability of the cross-links, which enables stress redistribution and energy dissipation through chain rearrangement within the polymer network.

#### 3.1.3. Multi-Axis Deformation

**Figure 13:**
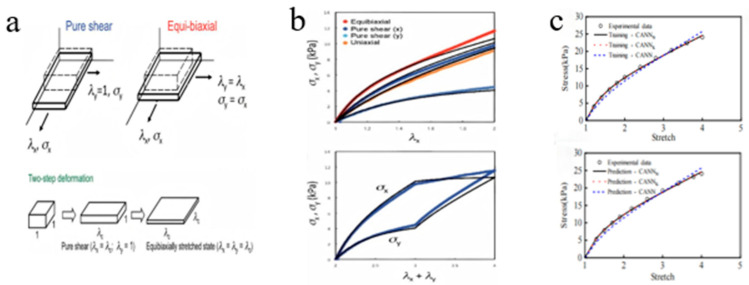
Multi-axis deformation. (**a**) Schematic diagram of loading scheme (Reproduced from Ref. [100] with permission from American Chemical Society). (**b**) Stress–strain response under uniaxial stretching, equal biaxial stretching, and two-step stretching (Reproduced from Ref. [100] with permission from American Chemical Society). (**c**) The stress–strain curves of the dehydrated SR gel were simulated using CANN_N_, PICANN, and CANN_K_ (Reproduced from Ref. [49] with permission from Wuhan University of Technology).

The mechanical response of CD-based SRPs under multiaxial stress conditions represents a crucial aspect for evaluating their potential as soft functional materials. To address this, researchers have employed experimental approaches including pure-shear and biaxial tensile testing (Figure 13a) to systematically investigate their distinctive deformation mechanisms.

Through various testing, modes including equibiaxial stretching (λ_x_ = λ_y_), pure-shear (λ_y_ = 1), where λ*_i_* (*i* = *x*, *y*) is the stretch along the *i*-axis, and two-step biaxial deformation [78,99,100,101,102], researchers observed that SR gels with low cross-linking density exhibit nearly ideal neo-Hookean elastic behavior, with minimal strain coupling between different directions (Figure 13b). These findings confirm that the free energy density function of low cross-linking density SR gels is independent of the second invariant (I_2_) of the strain tensor.

Considering the uniaxial tensile stress–strain behavior observed in experiments before and after dehydration, Li and Fu [49] developed a novel constitutive artificial neural network model (CANN_N_) based on Kuhl’s constitutive artificial neural network (CANN_K_) and physics-informed constitutive artificial neural network (PICANN) model for SRPs, which effectively captures the nonlinear mechanical response of SR gels. Their analysis revealed that as the hydration level of the gel changes, the strain energy function transitions from depending solely on I_1_ to depending on both I_1_ and I_2_ (Figure 13c). This shift indicates that the deformation mechanism of SR gels can be controllably altered through reversible dehydration and rehydration processes. It is further suggested that the underlying mechanism involves changes in chain entanglements induced by water loss or absorption. These entanglements act similarly to sliding cross-links during deformation, effectively modulating the effective cross-linking density of such dynamic networks. Thus, by varying the water content, the sliding cross-link density can be reversibly tuned, enabling active control over the gel’s deformation mechanism.

### 3.2. Fracture

To systematically elucidate the performance advantages of SRPs over conventional chemical gels typically involves combines of systematic experimentation with multi-scale characterization, with emphasis on fracture toughness evaluation. Standard uniaxial tensile testing serves as the primary method for quantitative toughness assessment, typically employing rectangular strip specimens tested on tensile testing machines at crosshead speeds ranging from 0.0083 mm/s to 2.68 mm/s [101,103]. Table 5 summarizes the data of sample size, type, and tensile rate in experimental studies.

The fracture properties of SRPs have been investigated using a variety of experimental methods and metrics to systematically assess their fracture energy and crack propagation behavior. Common approaches involve uniaxial tensile tests on samples with or without pre-cracked notches, where the fracture energy is determined using methods such as the J-integral or crack-tip opening displacement [103,104]. Advanced in situ observation techniques have provided direct evidence for understanding the fracture mechanisms of SRPs. It should be noted that standardized pure-shear tests and tear tests have not yet been systematically applied in the study of SR gels.

**Figure 14:**
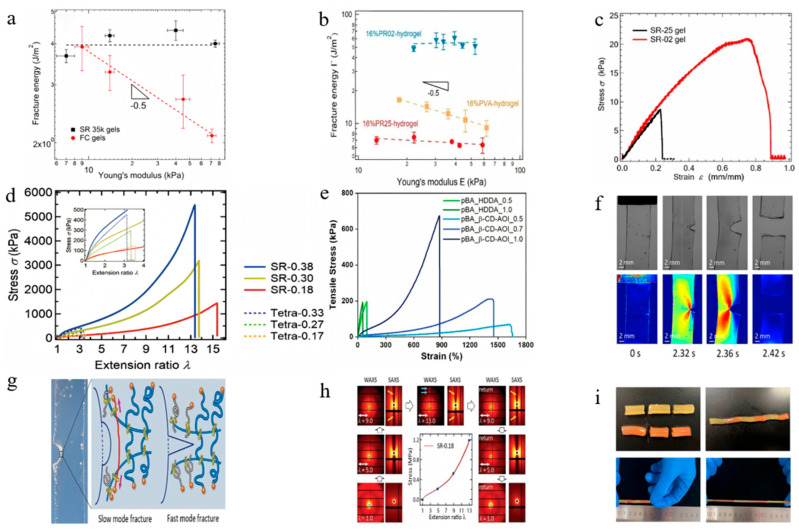
High fracture toughness of CD-based SRPs confirmed by fracture performance tests. (**a**) Curve of fracture energy and Young’s modulus of the SR gels and fixed cross-linking (FC) gels (Reproduced from Ref. [103] with permission from American Chemical Society). (**b**) Relationship between fracture energy and Young’s modulus in PVA, PR25, and PR02 hydrogels (Reproduced from Ref. [46] with permission from American Chemical Society). (**c**) Stress–strain curve of SR gels with different coverage rate (Reproduced from Ref. [101] with permission from The Electrochemical Society). (**d**) Stress–strain curve of SR gels and Tetra-arm (Tetra) gels with different volume fractions (Reproduced from Ref. [18] with permission from The American Association for the Advancement of Science). (**e**) Stress–strain curves of acrylic elastomers (pBA) with different cross-linking agents (Reproduced from Ref. [56] with permission from American Chemical Society). (**f**) Comparison of grayscale images and optical delay distribution of SR-25 gel during crack propagation (Reproduced from Ref. [101] with permission from The Electrochemical Society). (**g**) Schematics of the network behavior of SR gels during slow mode and fast mode fractures (Reproduced from Ref. [104] with permission from Elsevier). (**h**) SAXS and WAXS patterns of SR-0.18 gel during a loading–unloading cycle (Reproduced from Ref. [18] with permission from The American Association for the Advancement of Science). (**i**) Self-healing behavior of the PR-Gel (40 *w*/*v*%) at 25 °C (Reproduced from Ref. [19] with permission from Springer Nature).

Fracture performance studies have systematically evaluated the fracture energy and crack propagation behavior of SRPs using single-edge notched specimens. Experimental data demonstrate that the sliding cross-linked architecture significantly enhances fracture toughness. Liu et al. [103] reported fracture energies of approximately 40 J/m^2^ for SR gel with molecular weight of 35 kDa of PEG and coverage rate of 25% in uniaxial tensile tests (Figure 14a). Jiang et al. [46] measured fracture energies of 55 J/m^2^ for SR gel with coverage rate of 2%, substantially exceeding the 6 J/m^2^ observed for SR gel with coverage rate of 25% (Figure 14b). Liu et al. [101] determined a fracture energy of 28.8 J/m^2^ for SR gel with coverage rate of 2% (Figure 14c), while another study by Liu et al. [18] evaluated fracture energies up to 3600 J/m^2^ for SR gels with molecular weight of 38 kDa of PEG and coverage rate of 2% (Figure 14d). According to reference [18], the authors attribute the significant two-order-of-magnitude increase in fracture energy to the toughening mechanism of strain-induced crystallization by designing SR gel with low CD coverage, high PEG concentration and fully crosslinking. Yi et al. [56] reported fracture work of 1.98 MJ/m^3^ for SRPs cross-linking agent concentration of 1 w% without pre-cracks (Figure 14e), and Iijima et al. [65] obtained 360 MJ/m^3^ for dumbbell-shaped RCPs with DP*n* value of 70 for PTHF parts. The primary toughening mechanism involves the formation of an extensive damage zone ahead of the crack tip, where sliding cross-links effectively dissipate energy through mobility and rearrangement. Consequently, the fracture toughness of the material has been significantly enhanced. Li and Fu [49] prepared SR gels with different water loss rates through three different dehydration strategies and conducted tensile fracture experiments. The results showed that the fracture work of the dehydrated SR gel was 4.65 MJ/m^3^ (SR gel with water content of 24.75%). Table 6 summarizes the mechanical properties under uniaxial tensile fracture test.

Studies demonstrate that the fracture toughness of SRPs can be effectively optimized through control of their sliding cross-linked network parameters (PR concentration, cross-linking density). Increasing the PR concentration from 16% to 25% enhanced the fracture energy of SR gel with coverage rate of 2% from approximately 55 J/m^2^ to 157 J/m^2^ [46]. Furthermore, by optimizing the cross-linking density (achieved with CD coverage of 2%), SR gel with coverage rate of 2% maintain high ductility (exceeding 1600% elongation at break) while demonstrating fracture resistance over ten times greater than conventional chemical gels [46]. Li and Zhang [97] prepared SR gels with different coverage rates and cross-linking densities and conducted tensile fracture tests, measuring the SR gel with the coverage rate of 4.4% fracture work of 2.115 MJ/m^3^. These findings systematically establish that the mechanical properties of SRPs can be directionally enhanced through rational molecular structure design.

Characterization methods including high-speed polarized light imaging [101] and synchrotron radiation X-ray scattering [18] have enabled real-time monitoring of stress distribution and crack propagation (Figure 14f). These studies reveal that the sliding cross-linked architecture facilitates stress redistribution at crack tips, effectively retarding rapid crack advancement (Figure 14g) [104]. Liu et al. [103] prepared SR gels with varying crosslinker concentrations and compared them with FC gels. By combining the J-integral-crack tip opening displacement method with real-time crack propagation observation during uniaxial stretching, they systematically analyzed the crack propagation behavior. The experiments revealed that the tearing modulus of the SR gels was significantly higher than that of FC gels with similar Young’s modulus, indicating that sliding cross-links can effectively release stress concentration at the crack tip during slow crack growth. Furthermore, strain-induced crystallization [18] (Figure 14h) serves as a key mechanism enabling rapid self-reinforcement and high toughness in certain SRP systems. Liu et al. [18] prepared SR gels with different PEG volume fractions and compared them with conventionally cross-linked Tetra-PEG gels. By combining wide-angle X-ray scattering and small-angle X-ray scattering techniques during uniaxial tensile testing, the structural evolution of the gels under cyclic loading was monitored in real time. The experiments revealed that distinct diffraction spots emerged in the SR gels at large strains, indicating the occurrence of strain-induced crystallization of the PEG chains.

Self-healing capability enables SR gels to functionally recover after fracture. When fractured surfaces are brought into contact, CDs and guest molecules exposed on the surfaces can undergo thermally driven re-recognition and recombination, reestablishing the sliding cross-linked network and thereby achieving material self-repair. Xiong et al. [19] demonstrated the autonomous self-healing capability of their specially designed PR-Gel. The self-healing process was achieved by simply bringing two cylindrical gel specimens into contact at their cut interfaces and allowing them to stand at ambient temperature. The successfully self-healed gel withstood tensile loading without fracture during the demonstration, with mechanical strength recovering significantly within tens of seconds after the removal of high strain. Although the healing efficiency depends on factors such as temperature, time, and the host–guest binding constant, this material system achieved high-performance recovery without external intervention, highlighting the effectiveness of its molecular design.

### 3.3. Others

Swelling

**Figure 15:**
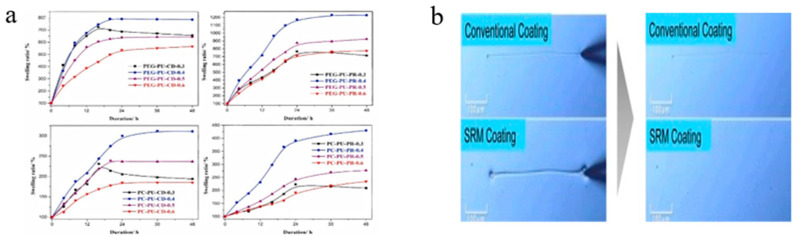
Swelling and scratch self-recovery. (**a**) Swelling curve of β-CD and β-CD [3] PR cross-linked polyurethanes (Reproduced from Ref. [84] with permission from Elsevier). (**b**) Scratch test of SRP coatings and conventional coatings (Reproduced from Ref. [105] with permission from John Wiley and Sons).

Sliding cross-linked networks significantly enhance transport properties within polymer materials. At equivalent cross-linking ratios, the mobility of cross-links confers substantially improved swelling capacity. Okumura et al. [90] prepared capillary topological gels that exhibited dry-weight expansion up to 24,000 times, far exceeding the approximately 2000 times swelling of conventional chemical gels. This behavior directly demonstrates the exceptional flexibility and expansibility of sliding cross-linked networks. Song et al.’s [84] swelling experiments showed that sliding cross-linked polyurethane films achieved higher (about 75%) equilibrium swelling ratios in dimethyl sulfoxide (Figure 15a). Okumura et al. [90] explained that an elevated the swelling ratio at cross-linking points leads to an enlargement of the mesh size within the network. The associated sliding capability further enables structural rearrangement of the network, which optimizes the diffusion pathways. As a result, solvent molecules can penetrate more rapidly, thereby enhancing the overall transport performance.

Self-recovery

Noda et al. [105,106] performed scratch tests on SRP coatings using needle tips and monitored the recovery process (Figure 15b). The results demonstrate that even at high cross-linking densities, SRPs retain exceptional elasticity and flexibility, enabling rapid rebound and scratch filling within seconds after deformation, thereby exhibiting highly efficient self-recovery capability. Noda et al. [105] explained that the self-recovery behavior observed in scratches of SRPs originates from their sliding cross-linked network architecture and constitutes a physical process driven by entropy increase. Fundamentally, the SRP employs its intrinsic capacity for sliding deformation to dissipate external mechanical stress. Through the inherent tendency of the system to maximize entropy, macroscopic damage is autonomously restored.

## 4. Deformation Mechanism and Theoretical Models of CD-Based SRPs

The deformation and fracture behavior of CD-based SRPs fundamentally transcends the predictions of conventional polymer network theories. Their macroscopic mechanical responses cannot be fully captured by classical frameworks. This section aims to provide a mechanistic analysis of the sliding deformation capacity in SRPs at the microscopic level, while systematically reviewing the constitutive models and fracture theories.

### 4.1. Hyperelastic Models

To establish the intrinsic relationship between macroscopic mechanical response and microscopic sliding deformation mechanisms in CD-based SRPs, researchers have developed various constitutive theories. Table 7 and Table 8 summarize the constitutive models that can be used for SRPs.

**Figure 16:**
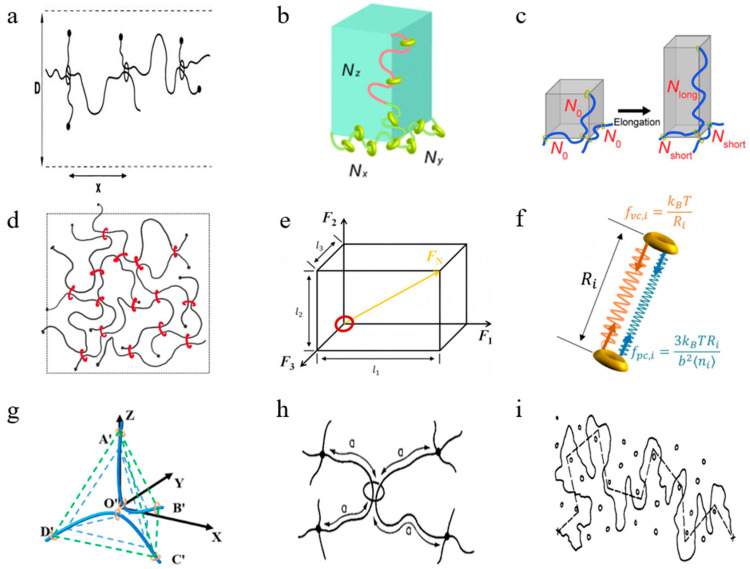
Schematic diagrams of different constitutive models. (**a**) de Gennes model (Reproduced from Ref. [20] with permission from Elsevier). (**b**) Mayumi–Ito model (Reproduced from Ref. [51] with permission from Royal Society of Chemistry). (**c**) 3-chain model (Reproduced from Ref. [108] with permission from American Chemical Society). (**d**) Vernerey–Lamont model (Reproduced from Ref. [109] with permission from Elsevier). (**e**) Non-affine model (Reproduced from Ref. [110] with permission from Royal Society of Chemistry). (**f**) Phantom slide-ring model (Reproduced from Ref. [111] with permission from American Chemical Society). (**g**) Sliding deformation 4-chain model (Reproduced from Ref. [97] with permission from Wuhan University of Technology). (**h**) Ball–Doi model (Reproduced from Ref. [118] with permission from Elsevier). (**i**) Edwards–Vilgis model (Reproduced from Ref. [119] with permission from Elsevier).

Early theoretical models primarily focused on sliding deformation. In 1999, de Gennes proposed a physical theory based on entropy and physical osmotic pressure, which addressed the softening behavior of slide-ring gels under low stress (Figure 16a) [20]. This theory represents a foundational contribution to the understanding of the mechanical behavior of SRPs. In 2005, Okumura et al. [90] incorporated sliding cross-links into the classical three-chain model, proposing an “extended 3-chain constitutive model” that successfully explained the J-shaped stress–strain curves, high extensibility, and hysteresis-free recovery of SR gels. In 2007, Ito et al. [107] proposed the free junction model to explain the sliding behavior within SR gels, particularly their unusual softness under low stress and high extensibility. This model idealizes the cross-linking points as nodes that slide freely along the polymer backbone, capable of accounting for the mechanical behavior of SR gels under various stress conditions.

Mayumi et al. [51] observed a non-monotonic dependence of the elastic modulus on cross-linking density. By modifying the three-chain model and incorporating the configurational entropy of cyclic molecules, they proposed the Mayumi–Ito model, which successfully explains this anomalous behavior (Figure 16b). Mayumi et al. [108], aiming to explain the unique mechanical property of SR gel, proposed a 3-chain model based on sliding cross-linking points, it effectively explains the phenomenon that the Young’s modulus of SR gel is lower than that of traditional fixed cross-linked gel. Vernerey et al. [109] developed the constitutive Vernerey–Lamont model based on transient network theory by incorporating internal variables characterizing chain sliding and stretching, successfully predicting transient responses including stress relaxation and energy dissipation behavior of SRPs (Figure 16d).

Lu et al. [110] established the cubic representative volume element (RVE) model by considering the sliding behavior of the ring on the polymer chain and the resulting molecular friction and then proposed the non-affine constitutive model (Figure 16e), successfully explaining the mechanism of high ductility and high toughness of the SR gel. Chen et al. [111] proposed the phantom slide-ring model (Figure 16f), representing network chains as parallel arrangements of pulley chains and virtual chains to quantitatively predict swelling dependence and nonlinear strain softening of the SR gel.

Xing et al. [112] established the spontaneous equilibrium free energy model for the sliding deformation and sag effect in SRPs, quantitatively analyzed the sliding and sag behavior of the chain, and was able to accurately predict the mechanical behavior of SRPs under different loading conditions.

Regarding the geometric characteristics of figure-of-eight shaped sliding cross-link configurations, Li and Zhang [97] proposed a sliding deformation 4-chain model (Figure 16g) for the hyperelastic large deformation of CD-based SRPs by introducing sliding cross-link points into the traditional 4-chain model. Numerical experiments have shown that, compared with traditional hyperelastic constitutive models such as the neo-Hookean model, Yeoh model, and the Arruda–Boyce model, the sliding deformation four-chain model can better fit the uniaxial tensile stress–strain curves of SR gels with different coverage rates and can describe the stress softening behavior of SR gels at large strains.

In a pioneering study, Li and Fu [49] introduced artificial neural networks into the research of SR gels. Building upon Kuhl’s constitutive artificial neural network (CANN_K_) framework, they developed a physics-informed constitutive artificial neural network (PICANN) model for SR materials. This model was designed to predict the stress–strain curves of SR gels under uniaxial tension, equibiaxial tension, and two-step stretching, considering different synthesis parameters, namely, the cross-linking agent concentration and the concentration of PR. The PIACNN and MPNN (PICANN × MPNN) model represents an improvement over PICANN by incorporating the physical insight that the free energy function of SRPs depends solely on the first strain invariant I_1_, enabling it to accurately capture the stress–strain response under biaxial loading. Furthermore, a new constitutive artificial neural network (CANN_N_) model was developed to predict the uniaxial tensile stress–strain curves of SR gels at different water contents by rationally constructing a convex coupling term of the first and second strain invariants, I_1_ and I_2_, into the free energy function and using the experimental data from uniaxial tensile tests as training and testing datasets for model development and validation. By fitting the uniaxial tensile stress–strain curves of specimens with varying water contents, it was discovered that the deformation mode of the SR gel transitions from being dependent on both I_1_ and I_2_ at low water content to being dependent solely on I_1_ at high water content, and this process is reversible. This suggests that dehydration induces entanglement of the polymer chains, where the sliding of cyclodextrins is constrained or even completely immobilized by these entanglements, effectively driving the transition of SR gels toward chemically cross-linked gels.

Bitoh et al. [100] demonstrated through systematic biaxial testing that low cross-linking density SR gels exhibit nearly neo-Hookean stress–strain behavior under different biaxial loading, revealing their distinctive minimal strain-coupling characteristic. Kondo et al. [113] revealed that the strain energy density function of SR gels under unequal biaxial deformation depends solely on the first invariant of the strain tensor. Their analysis demonstrated that the Gent model successfully captures this distinctive nonlinear elastic response, indicating a significantly weakened explicit coupling effect between strains in different directions for the SR gels. Kato et al. [114] addressed the anomalous decrease in the elastic modulus of SR gels at high cross-linking densities by proposing a “gas-like” entropic pressure model for the cyclic molecules, which was integrated with the neo-Hookean constitutive relation. Yun and Kim [115] demonstrated that slide-ring elastomers follow neo-Hookean stress–strain behavior, and the sources of their high flexibility and toughness were explained.

Xing et al. [116], by introducing the topological junction theory and the extended tube model, proposed the Xing–Shu model, which successfully quantitatively described the complex interactions between molecular chains and cross-linked rings in SR gels, solving the balance problem between ductility and toughness of traditional gels.

Li and Wei [117] were the first to propose the use of the Ball–Doi model (Figure 16h) [118] and the Edwards–Vilgis model (Figure 16i) [119] for simulating the large deformation and fracture behavior of sliding cross-linked polymers, as well as the first to apply the Mayumi–Ito model and the Vernerey–Lamont model to simulate the fracture behavior of SR polymers. In ABAQUS finite element simulation and non-ordinary state-based periodic dynamics (NOSB-PD) simulation, four hyperelastic constitutive models were used to simulate the uniaxial tensile tests and single-edge crack uniaxial tensile fracture tests of SR gels with different coverage rates and volume fractions. The numerical results demonstrated that the Edwards–Vilgis model exhibits good applicability for SR gels with a coverage rate of 2% under large tensile deformation. In the uniaxial tensile fracture simulations, both the Ball–Doi model and the Edwards–Vilgis model showed strong applicability under 2% coverage, consistent with the observed mechanical responses.

**Table 8:** Other constitutive models used for SR gels.

**Constitutive Model**	**Function Expression**
neo-Hookean model [100,114,115]	$W=\frac{G}{2}(\lambda_{x}^{2}+\lambda_{y}^{2}+\lambda_{z}^{2}-3)$
Gent model [113]	$W=-\frac{G}{2}(I_{m}-3)\ln(1-\frac{I_{1}-3}{I_{m}-3})$
Xing–Shu model [116]	$W=N_{s}k_{B}T(\ln\frac{h}{h_{0}}-2\ln\frac{h^{2}-r_{\max}^{2}}{h_{0}^{2}-r_{\max}^{2}})$
Ball–Doi model [118]	W=12NckBT∑λ2+i12NskBT∑λi(21+η)1+ηλi2+12NskBT∑log(1+ηλ2)i
Edwards–Vilgis model [119]	W=12NckBT∑λ2(i1−α2)1−α2∑λi2+log(1−α2∑λ2)i+12NskBT∑λ2(i1−α2)(1+η)(1−α2∑λ2)i(1+ηλ2)i+log(1+ηλ2)i+log(1−α2∑λ2)i

Note: *W*: free energy; *N_s_*: number of solvent molecules in the gel; *k_B_*: Boltzmann constant; *T*: absolute temperature; *N_c_*: number of elastically effective chains in the network; *λ_i_*: principal elongation ratios; *G*: shear modulus; *I*_1_: first strain invariant; *I_m_*: limiting strain invariant; *h*: end-to-end distance of stretched chains; *h*_0_: initial end-to-end distance; *r*_max_: maximum sliding distance; *η*: measure of sliding freedom; α: measure of inextensibility.

Of course, these constitutive models can also be categorized into four main types: early models based on phenomenology/entropy (such as the free junction model [107], the neo-Hookean model [100,114,115]), chain-network-based analytical models (such as the extend 3-chain model [90], the 3-chain model [108], the sliding deformation 4-chain model [97]), non-affine and friction-based models (such as the non-affine model [110], the phantom slide-ring model [111]), and machine-learning and data-driven models (such as the PICANN × MPNN model [49], the CANN_N_ model [49]).

### 4.2. Fracture Models

**Figure 17:**
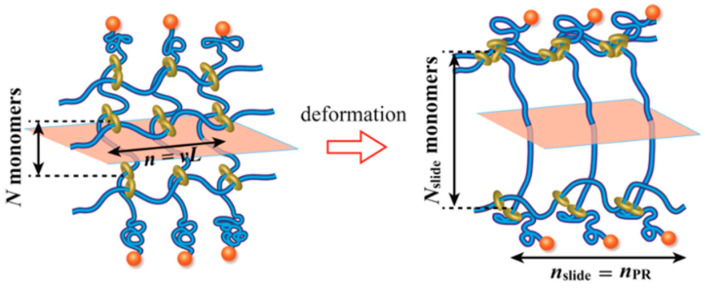
Liu–Kadono model for SR gels (Reproduced from Ref. [103] with permission from American Chemical Society).

Fracture modeling aims to elucidate the fundamental mechanisms underlying the high fracture energy and fatigue thresholds of SRPs. Early research primarily focused on their exceptional fracture toughness under quasi-static loading conditions. Table 9 summarize the fracture models used for SR gels. Based on the Lake–Thomas theory, Liu et al. [103] proposed the Liu–Kadono model to address the inability of the traditional Lake–Thomas model to explain the fracture energy of SR gels (Figure 17). The core of this model lies in its consideration of the crucial factor of the sliding range of the cross-links. When calculating the fracture energy, it employs the network structure parameters in the fully deformed state rather than those in the initial state. Jiang et al. [46] established the Jiang–Liu model based on the Lake–Thomas theory to explain the anomalous phenomenon that the fracture energy of the low-coverage SR gel is independent of the Young’s modulus. The core of this model lies in considering the influence of the slide ability of the slide-ring cross-linking points on the network structure during the fracture process. This model successfully links macroscopic mechanical properties (high fracture energy) with microscopic molecular mechanisms (sliding deformation). It is clarified that by increasing the sliding range of the axial polymer (for example, using PEG with a higher molecular weight), gel materials with extremely high fracture toughness can be effectively designed and prepared without significantly sacrificing stiffness. To explain the significant toughness reduction observed during high-speed stretching, Liu et al. [120] established a Liu–Yokoyama model combining Lake–Thomas theory with molecular dynamics of sliding cross-links, successfully describing the transition from sliding-dominated to fixed cross-link-dominated fracture behavior.

### 4.3. Viscoelastic Models

Beyond constitutive models simulating fundamental mechanical behavior, researchers have developed viscoelastic constitutive models for SRPs, significantly advancing the understanding and application potential of this material system. Table 10 summarizes viscoelastic constitutive models used for SR gels.

Qian et al. [121] proposed a novel slide-ring adhesion system based on a movable cross-linked network. The performance of the material was analyzed using the Kohlrausch–Williams–Watts (KWW) model and the Burgers model. It has been quantitatively proven that the sliding cross-linked structure combines rapid energy dissipation with long-term creep resistance. Dikshit and Bruns [122] employed chemical rheology combined with small-amplitude oscillatory shear to track the evolution of storage and loss moduli, establishing a phenomenological dynamic model based on a modified Hill equation. This framework quantitatively describes how cross-linking rate depends on temperature, cross-linker concentration, and quasi-rotaxane concentration, while revealing the direct correlation between cross-linking density and plateau modulus, thereby providing a basis for tailoring SR gel mechanical properties.

## 5. Simulation and Prediction of CD-Based SRPs

The advancement of experimental characterization and theoretical modeling has significantly enhanced our comprehension of the macroscopic mechanical behavior of CD-based SRPs. However, computational simulations are important for establishing precise structure–property relationships at the molecular scale and ultimately achieving targeted design of high-performance materials. This chapter systematically reviews progress in advanced approaches to SRP research progress in SRPs, encompassing simulation methodologies across atomic and continuum scales, focusing particularly on the role and contributions of various simulation methods in analyzing the microscopic mechanisms of “sliding cross-linking”, and predicting macroscopic material properties.

### 5.1. Simulation at Atomic or Molecular Scale

Atomic and molecular-scale simulations provide crucial insights into the microscopic mechanisms of sliding cross-linking, with the primary objective of investigating CD motion along polymer chains to establish theoretical foundations for understanding macroscopic material properties.

**Figure 18:**
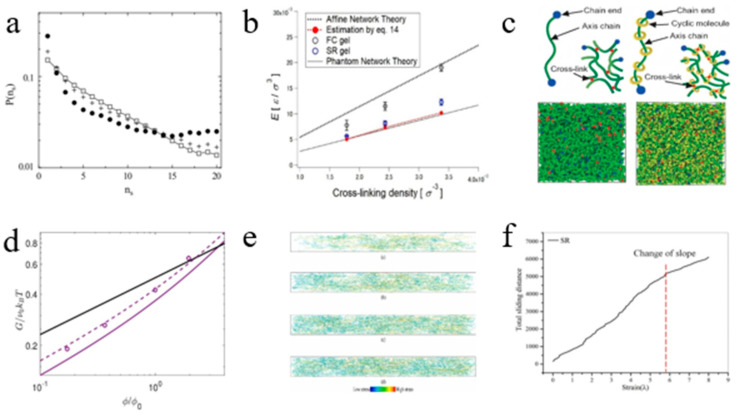
Simulation of SRPs at the atomic or molecular scale. (**a**) The relationship between the distribution of subchain lengths and the stretch ratio (Reproduced from Ref. [123] with permission from Springer Nature). (**b**) The cross-linking density dependence of the Young’s modulus of the FC gel and the SR gel (Reproduced from Ref. [108] with permission from American Chemical Society). (**c**) Coarse-grained models of SR gels and fixed cross-linked gels (Reproduced from Ref. [124] with permission from American Chemical Society). (**d**) Curve of normalized shear modulus varying with volume fraction (Reproduced from Ref. [111] with permission from American Chemical Society). (**e**) Adding end-groups can transform the failure of entire segments into piecewise failed (Reproduced from Ref. [47] with permission from Elsevier). (**f**) Total sliding distance of the slide ring in the SR gels during the stretching process (Reproduced from Ref. [79] with permission from Elsevier).

Koga and Tanaka [123] used Brownian Dynamics (BD) to preliminarily validate the aggregation behavior of sliding cross-links and the reduction in effective chain number during stretching, offering initial physical interpretation of the “pulley effect” (Figure 18a). However, such models exhibited significant simplifications, neglecting actual CD chemical structures, solvent effects, and non-Gaussian hardening from finite chain extensibility, while also lacking clear parameter correspondence with experimental systems. Yasuda et al. [108] employed coarse-grained molecular dynamics simulations within LAMMPS to conduct uniaxial stretching tests on slide-ring hydrogels. Their work quantitatively correlates the sliding activity of cross-links with the material’s Young’s modulus, thereby addressing the limitation of traditional theories in describing the influence of cross-link sliding on mechanical properties (Figure 18b). Uehara et al. [124] constructed a polymer model incorporating slide-ring structures via coarse-grained molecular dynamics (Figure 18c), comparing fracture behaviors between SR gels and conventional chemical gels to reveal the distinctive fracture mechanisms of SR gels under tensile deformation. Chen et al. [111] employed molecular dynamics simulations with a bead-spring model to construct slide-ring hydrogel networks, performing equilibrium and deformation calculations in LAMMPS. The resulting elastic modulus and stress–strain relationships aligned with predictions from the “phantom slide-ring” theoretical model (Figure 18d). Their research demonstrates that chain segment redistribution and monomer number per chain collectively determine the material’s low modulus, high ductility, and uniform stress distribution. Li and Liu [47,125] established a coarse-grained molecular dynamics model of SR gels to investigate the global failure mechanism arising from end-group constrained CD sliding in long chains (Figure 18e). Through tensile and fracture simulations in LAMMPS, they demonstrated that introducing stopper groups transforms “whole-chain fracture” into “segmented failure,” significantly enhancing long-term durability. Li and Tang [79] conducted coarse-grained molecular dynamics simulations to systematically investigate the mechanical responses of three distinct gel systems—SR gels, FC gels, and physical cross-linking (PC) gels. The study encompassed uniaxial tensile, static fatigue, and dynamic fatigue tests, performed using the LAMMPS simulation package (Figure 18f). Their work clarified that the “strength-toughness trade-off” originates from limitations in maximum sliding distance, providing theoretical guidance for addressing insufficient strength in SR gels.

### 5.2. Simulation at Continuum Scale

Continuum-scale simulation serves as an indispensable tool for investigating the macroscopic mechanical behavior of SRPs. By employing constitutive models that incorporate sliding deformation, this approach directly predicts the nonlinear stress response and fracture phenomena of SRPs.

**Figure 19:**
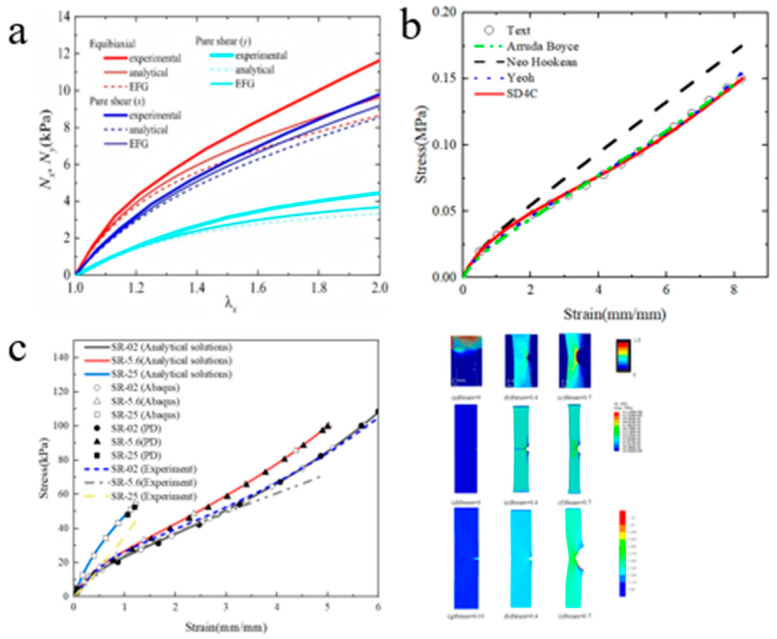
Mechanical response under continuum medium scale simulation. (**a**) stress–strain curves under multiaxial deformation (Reproduced from Ref. [126] with permission from Springer Nature). (**b**) Stress–strain curves of different constitutive models under uniaxial tension (Reproduced from Ref. [97] with permission from Wuhan University of Technology). (**c**) Stress–strain curves and fracture process diagrams of the Edwards–Vilgis constitutive model under uniaxial stretching (Reproduced from Ref. [117] with permission from Wuhan University of Technology).

Conventional element-based numerical methods, such as the finite element method, may encounter challenges like mesh distortion when simulating extremely large deformations. Li et al. [126] applied the element-free Galerkin method to the complex large deformation analysis of SR gels for the first time (Figure 19a).

Li and Zhang [97] were the first to simulate the large deformation tensile behavior of SR gels based on their sliding deformation 4-chain model. By implementing the model into the UHYPER subroutine of ABAQUS, they validated the model’s accuracy through comparisons of numerical solutions with uniaxial tensile experimental data of SR gels with different coverage rates (Figure 19b).

Li and Wei [117] conducted the first continuum-scale simulation of large deformation fracture in SR gels. Two numerical schemes were employed: finite element analysis via ABAQUS and non-ordinary state-based peridynamics (NOSB-PD). In the ABAQUS simulations, cohesive elements were utilized to model Mode I crack propagation in the SRPs, while in the NOSB-PD approach, fracture was captured by introducing a critical bond stretch criterion. Numerical experiments were performed using four hyperelastic constitutive models to simulate both uniaxial tension and single-edge notch tensile fracture tests on SR gels with varying coverage ratios and volume fractions. The results demonstrated that fracture simulations under different coverage ratios using the NOSB-PD method showed the best agreement with experimental data under 25% coverage when employing the Vernerey–Lamont constitutive model. For fracture simulations across different volume fractions using NOSB-PD, all three constitutive models exhibited progressively improved fitting accuracy as the volume fraction decreased (Figure 19c).

## 6. Conclusions

This review systematically examines recent advances in structural design, performance characterization, and mechanistic understanding of CD-based SRPs. Studies demonstrate that SR gels incorporating “figure-of-eight” cross-links constitute the most thoroughly investigated system; however, research on their macroscopic mechanical behavior has predominantly focused on tensile and fracture properties, with a systematic experimental strategy still lacking. While numerous constitutive models have been applied to SR gels, most remain phenomenon-specific, and a universal theoretical framework capable of fully capturing their complex nonlinear mechanical responses has not yet been established. Computational investigations in this domain also remain relatively underdeveloped. For other sliding cross-linked polymer systems based on alternative topological configurations, mechanical characterization remains largely restricted to tensile testing, with corresponding theoretical modeling still in its nascent stages. The critical structure–property relationships connecting molecular mechanisms to macroscopic performance await systematic establishment. Future research should prioritize advanced experimental investigations under complex loading conditions, development of universally applicable theoretical frameworks, and enhanced multi-scale computational simulations to achieve transformative progress in this field.

## Figures and Tables

**Table 1:** Molecular configuration of cross-linking agents and their cross-linking points.

Cross-Linking Agent	Cross-Linking Configuration	References
*N*,*N′*-carboxyldiimidazole (CDI)	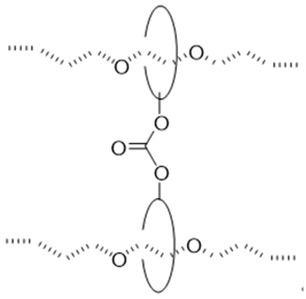	[50,51]
1, 4-butanediol diglycidyl ether (BDDE)	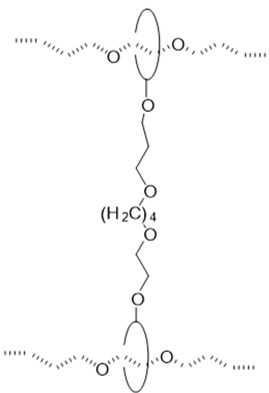	[52]
Divinylsulfone (DVS)	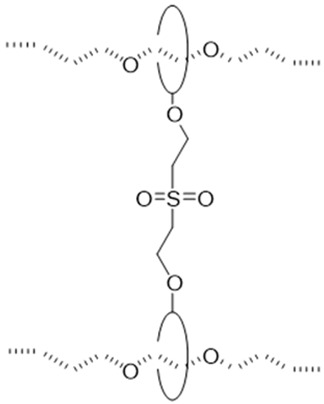	[46]
Hexamethylene diisocyanate (HMDI)	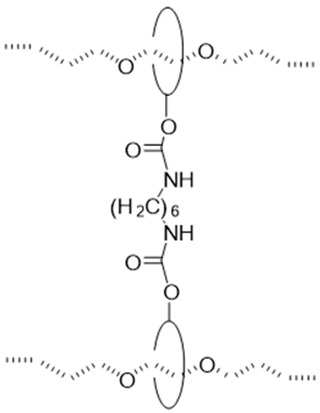	[53,54,55]

**Table 5:** Uniaxial tensile fracture test.

Experimental Conditions	
Rectangular strip	25 × 10 × 1 mm^3^ [46] (2 mm single-edge notch) 40 × 10 × 1 mm^3^ [101,103] (2 mm single-edge notch) 15 × 4 × 1 mm^3^ [18] (1 mm single-edge notch) 30 × 3 × 1 mm^3^ [49,97] (without notch)
Dumbbell—shaped	15 × 10 ×1 mm^3^ [56] (without notch) 12 × 2 × 0.68–0.94 mm^3^ [65] (without notch)
Rate	0.0083 mm/s [94], 0.167 mm/s [65], 0.2 mm/s [103], 1 mm/s [46,56], 1.875 mm/s [18], 1.67 mm/s [49,97], 2.68 mm/s [101].

**Table 6:** Fracture toughness of CD-based SRPs.

Fracture Toughness	
Fracture energy	28.8 J/m^2^ (SR gel with coverage rate of 2%) [101]. 40 J/m^2^ (SR gels with molecular weight of 35 kDa of PEG and coverage rate of 25%) [103]. 55 J/m^2^ (SR gel with coverage rate of 2%) [46]. 3600 J/m^2^ (SR gels with molecular weight of 38 kDa of PEG and coverage rate of 2%) [18].
Fracture work	1.87 MJ/m^3^ (SRPs with cross-linking agent concentration of 1 w%) [56]. 2.115 MJ/m^3^ (SR gel with coverage rate of 4.4%) [97]. 4.65 MJ/m^3^ (SR gel with water content 24.75%) [49]. 16 MJ/m^3^ (SRPs mixed with HDI and MDI in an 80:20 ratio) [94]. 360 MJ/m^3^ (RCPs with DP*n* value of 70 for PTHF parts) [65].

**Table 7:** Constitutive models proposed for SRPs.

Constitutive Model	Function Expression
de Gennes model [20]	$W=k_{B}T(3\ln\frac{x}{a}+\frac{N^{2}v}{2D^{2}x^{2}})$
Extend 3-chain model [90]	$\sigma =vR_{0}f_{s}(1-\lambda^{-1.5})$
Free junction model [107]	$W=\frac{n}{2}k_{B}T[\ln\frac{N_{z}{\left(3N-N_{z}\right)}^{2}}{4}+N(\frac{\lambda^{2}}{N_{z}}+\frac{4}{\lambda (3N-N_{z})})]$
Mayumi–Ito model [51]	$\begin{array}{cc} W & =N_{c}k_{B}T\sum_{i}[\frac{3}{2}\lambda_{i}^{2}\frac{N}{N_{i}\left(\lambda^{\to }\right)}+\frac{3}{2}\ln N_{i}\left(\lambda^{\to }\right)-N_{i}\left(\lambda^{\to }\right)\ln N_{i}\left(\lambda^{\to }\right)+\\ & \left(N_{i}\left(\lambda^{\to }\right)-n)\ln(N_{i}\left(\lambda^{\to }\right)-n)+lnn!\right] \end{array}$
3-chain model [108]	$E_{SR}=E_{affine}(1-\frac{N_{slide}}{N_{0}})$
Vernerey–Lamont model [109]	$W=cNk_{B}T[\frac{1}{2}I_{1}+h\left(n_{0}\alpha \right)+\frac{2}{N}h(\frac{n_{t}-Nn_{0}\alpha }{2})]+\prod (\det(F)-1)$
Non-affine model [110]	$W=\frac{N}{3}(\delta e_{1}+\delta e_{2}+\delta e_{3})+k_{B}T(C\log\frac{\Omega C}{1+\Omega C}+\frac{\chi C}{1+\Omega C})$
Phantom slide-ring model [111]	$W=\frac{1}{2}[\frac{3k_{B}T}{2Nb^{2}}{\left(\sum_{i=1}^{q}\left|R_{i}\right|\right)}^{2}+k_{B}T\sum_{i=1}^{q}\ln\left|R_{i}\right|]$
Spontaneous equilibrium free energy model [112]	$\begin{array}{cc} W & =\frac{3N_{el}k_{B}T}{2}[\ln\frac{3\Omega_{0}}{2\pi b^{2}}+2-\ln{\left(\frac{\left(l_{t}^{2}\Lambda^{2}-1)(1+l_{t}^{2}\Lambda^{2}/2\right)}{l_{t}^{2}\Lambda^{2}}\right)}^{2}-l_{t}^{2}\Lambda^{2}]\\ & +\frac{3N_{0}k_{B}T}{2}\Lambda^{2}-N_{0}k_{B}T\frac{\alpha b^{2}}{3d_{r}^{2}}\sum \frac{1}{\lambda_{i}} \end{array}$
Sliding deformation 4-chain model [97]	$W=\frac{1}{2}NK_{B}T\left(I_{1}-3\right)\left(\frac{1-\lambda_{s1}}{1-\lambda_{s1}e^{-SI_{1}}}+\frac{1+\lambda_{s2}}{1+\lambda_{s2}e^{-SI_{1}}}-1\right)+\frac{1}{D_{1}}{\left(J-1\right)}^{2}$
PICANN × MPNN model [49]	$\begin{array}{cc} W & =\{\omega_{2,1}\omega_{1,1}[I_{1}-3]+\omega_{2,2}[\exp(\omega_{1,2}[I_{1}-3]-1]\\ & +\omega_{2,3}\omega_{1,3}{\left[I_{1}-3\right]}^{2}+\omega_{2,4}[\exp(\omega_{1,4}{\left[I_{1}-3\right]}^{2}-1]\}G\\ G & =\omega_{2,1}\omega_{1,1}c_{x}+\omega_{2,2}\omega_{1,2}c_{x}^{2}+\omega_{2,3}\exp(\omega_{1,3}c_{x})+\omega_{2,4}\omega_{1,4}c_{x}\ln(\omega_{1,4}c_{x})\\ & +\omega_{2,5}\omega_{1,5}c_{PR}+\omega_{2,6}\omega_{1,6}c_{PR}^{2}+\omega_{2,7}\exp(\omega_{1,7}c_{PR})+\omega_{2,8}\omega_{1,8}c_{PR}\ln(\omega_{1,8}c_{PR}) \end{array}$
CANN_N_ model [49]	$\begin{array}{cc} W & =\omega_{2,1}\omega_{1,1}[I_{1}-3]+\omega_{2,2}[\exp(\omega_{1,2}[I_{1}-3]-1]\\ & +\omega_{2,3}\omega_{1,3}{\left[I_{1}-3\right]}^{2}+\omega_{2,4}[\exp(\omega_{1,4}{\left[I_{1}-3\right]}^{2}-1]\\ & +\omega_{2,5}\omega_{1,5}[I_{2}-3]+\omega_{2,6}[\exp(\omega_{1,6}{\left[I_{2}-3\right]}^{2}-1]\\ & +\omega_{2,7}\omega_{1,7}{\left[I_{1}-3\right]}^{2}+\omega_{2,8}[\exp(\omega_{1,8}{\left[I_{1}-3\right]}^{2}-1] \end{array}$

Note: *W*: free energy density; *k_B_*: Boltzmann constant; *T*: absolute temperature; *x*: distance between two sliding cross-links; *a*: polymer chain length; *N*: number of monomers per polymer chain; *v*: excluded volume parameter; *D*: tube diameter; *σ*: stress; *υ*: effective chain density in the network; *f_z_*: tension of a single polymer chain in the stretching direction; *λ*: elongation ratio; *n*: total number of chain segments between cross-links; *N_z_*: number of monomers in the chain segment along the z-direction; *N_c_*: number of cross-links; *λ_i_*: principal elongation ratios in respective directions; *N_i_*(λ⃗): number of monomers in chain segments along each direction after deformation; *E_SR_*: Young’s modulus; *E_affine_*: Young’s modulus predicted by classical rubber elasticity theory; *N_slide_*: degree of sliding; *n*_0_: average number of monomers per network segment in the undeformed state; *c*: number density of PR chains; *I*_1_: first invariant; α: internal state variable; *n_t_*: total number of monomers in one PR chain; Π: Lagrange multiplier; F: deformation gradient; *δe_i_*: virtual work done by chain force on displacement; *C*: molar concentration of solvent; Ω: molar volume of solvent; *χ*: interaction parameter; *b*: statistical segment length; *q*: number of segments on the main chain; *R_i_*: end-to-end vector of the i-th segment; *N_el_*: number of elastic chains per unit volume participating in the pulley effect; Ω_0_: normalized Gaussian distribution parameter; *l_t_*: constraint parameter; Λ: micro–macro chain stretch ratio; *N*_0_: number of unconstrained chains per unit volume; *d_r_*: ring diameter; *λ_s_*_1_, *λ_s_*_2_: maximum sliding parameters introduced; *S*: coefficient relating sliding to stretch ratio; *D*_1_: incompressibility term coefficient; ω*_i_*_,*j*_: weight coefficient of the neural network; G: MPNN equation.

**Table 9:** Fracture models for SR gels.

Model	Function Expression
Liu–Kadono model [103]	$\Gamma_{SR}=n_{slide}N_{slide}U$
Jiang–Liu model [46]	$\Gamma_{PR}=R_{PR}c_{PR}N_{PR}U$
Liu–Yokoyama [120]	$\Gamma_{SR}=N_{slide}\left(\left(\sqrt{6}R_{g(PR)}U\rho \varphi \right)/M_{PR}\right)$

Note: Γ*_SR_*: fracture energy of slide-ring gel; *n_slide_*: number of PR chains per unit volume; *N_slide_*: number of slidable monomers per chain; *U*: energy required to break a single monomer; *R_PR_*: root-mean-square end-to-end distance of PR chain; *c_PR_*: number density of PR chains; *N_PR_*: number of slidable monomers between two cross-links per PR chain; *M_PR_*: molecular weight of PR; *R_g (PR)_*: radius of gyration of PR chain; *ρ*: polymer density; *φ*: volume fraction of polymer in the gel.

**Table 10:** Viscoelastic models for SR gels.

Viscoelastic Models	Function Expression
KWW model [121]	$\sigma =\sigma_{r}\exp\{-{\left(\frac{t}{\tau }\right)}^{\beta }\}+\sigma_{\infty }$
Burgers model [121]	$\varepsilon (t)=\frac{\sigma }{\eta_{1}}t+\frac{\sigma }{E_{2}}[1-\exp(-\frac{tE_{2}}{\eta_{2}})]$
Modified Hill model [122]	$G^{\prime }(t)=G_{\infty }^{\prime }\frac{t^{\alpha }}{t^{\alpha }+\theta^{\alpha }}$

Note: *σ*: Stress at time t; *σ_r_*: relaxable stress component; *t*: total time; *τ*: relaxation time; *β*: stretching exponent; *σ*_∞_: residual stress; *ε*(*t*): strain at time t; *η_1_*: steady-state creep viscosity; *E_2_*: spring modulus; *η_2_*: viscoelastic damping coefficient; *G′(t)*: storage modulus at time t; *G′_∞_*: plateau modulus; *α*: exponent fitting parameter; *θ*: time at which gelation reaches half-completion.

## Data Availability

No new data were created or analyzed in this study. Data sharing is not applicable to this article.

## Abbreviations

The following abbreviations are used in this manuscript:

CD	Cyclodextrin
β-CD	β-Cyclodextrin
γ-CD	Gamma-cyclodextrin
CCPs	Covalently cross-linked polymers
PCPs	Physically cross-linked polymers
RCPs	Rotaxane cross-linked polymers
SRPs	Slide-ring polymers
PEG	Polyethylene glycol
PPO	Polypropylene oxide
PTHF	Polytetrahydrofuran
PR	Polyrotaxane
TBMs	Terminal bulky macromonomers
VSC	Vinylic supramolecular cross-linkers
VC11	Viologen with C11 alkyl chain
DMF	Dimethylformamid
CDI	*N*,*N′*-carbonyldiimidazole
BDDE	1,4-butanediol diglycidyl ether
DVS	Divinyl sulfone
HMDI	Hexamethylene diisocyanate
MDI	Diphenylmethane diisocyanate
αCD-Azo	α-Cyclodextrin-Azo
AM	Acrylamide
MA	Methacrylate
KP	Knitted polymer
PVA	Polyvinyl alcohol
PNIPA	Poly(N-isopropylacrylamide)
SR	Slide-ring
FC	Fixed cross-linking
PC	Physical cross linking
Tetra	Tetra-arm
PR-Gels	Polymerizable rotaxane gels
RVE	Representative volume element
DMA	Dynamic mechanical analysis
SAOS	Small-amplitude oscillatory shear
NOSB-PD	Non-ordinary state-based peridynamics
BD	Brownian Dynamics
ML	Machine learning
CGMD	Coarse-Grained Molecular Dynamic
CANN_K_	Kuhl’s constitutive artificial neural network
PICANN	Physics-informed constitutive artificial neural network
PICANN × MPNN	PICANN and MPNN
CANN_N_	Novel constitutive artificial neural network model
KWW	Kohlrausch–Williams–Watts
